# Delivering genes across the blood-brain barrier: LY6A, a novel cellular receptor for AAV-PHP.B capsids

**DOI:** 10.1371/journal.pone.0225206

**Published:** 2019-11-14

**Authors:** Qin Huang, Ken Y. Chan, Isabelle G. Tobey, Yujia Alina Chan, Tim Poterba, Christine L. Boutros, Alejandro B. Balazs, Richard Daneman, Jonathan M. Bloom, Cotton Seed, Benjamin E. Deverman

**Affiliations:** 1 Stanley Center for Psychiatric Research, Broad Institute of MIT and Harvard, Cambridge, MA, United States of America; 2 Ragon Institute of MGH, MIT, and Harvard, Cambridge, MA, United States of America; 3 Departments of Neurosciences and Pharmacology, University of California, San Diego, La Jolla, CA, United States of America; 4 Department of Pharmacology, University of California, San Diego, La Jolla, CA, United States of America; National Institutes of Health, UNITED STATES

## Abstract

The engineered AAV-PHP.B family of adeno-associated virus efficiently delivers genes throughout the mouse central nervous system. To guide their application across disease models, and to inspire the development of translational gene therapy vectors for targeting neurological diseases in humans, we sought to elucidate the host factors responsible for the CNS tropism of the AAV-PHP.B vectors. Leveraging CNS tropism differences across 13 mouse strains, we systematically determined a set of genetic variants that segregate with the permissivity phenotype, and rapidly identified LY6A as an essential receptor for the AAV-PHP.B vectors. Interfering with LY6A by CRISPR/Cas9-mediated *Ly6a* disruption or with blocking antibodies reduced transduction of mouse brain endothelial cells by AAV-PHP.eB, while ectopic expression of *Ly6a* increased AAV-PHP.eB transduction of HEK293T and CHO cells by 30-fold or more. Importantly, we demonstrate that this newly discovered mode of AAV binding and transduction can occur independently of other known AAV receptors. These findings illuminate the previously reported species- and strain-specific tropism characteristics of the AAV-PHP.B vectors and inform ongoing efforts to develop next-generation AAV vehicles for human CNS gene therapy.

## Introduction

With the boom of gene replacement, knockdown, and editing technologies, the number of diseases that are potentially treatable by gene therapy is rapidly expanding. AAV vectors are proving to be safe, versatile vehicles for *in vivo* gene therapy applications [1–4]. However, delivery challenges impede the application of gene therapy, particularly in the context of the brain, which is protected by the blood-brain barrier (BBB). To improve gene delivery across the central nervous system (CNS), our group and others have engineered AAV capsids using *in vivo* selection and directed evolution [5–8]. Our group has focused on engineering AAV9 variants, such as AAV-PHP.B [6] and its further evolved, more efficient variant, AAV-PHP.eB [5], to cross the adult BBB and enable efficient gene transfer to the CNS. Since their invention, AAV-PHP.B and AAV-PHP.eB have been applied across a wide range of neuroscience experiments in mice [5,9,10], including genetic deficit correction [11,12] and neurological disease modeling [13].

A critical question has been how the AAV-PHP.B vectors cross the BBB and whether this mechanism can be translated to other species and, ultimately, humans. The enhanced CNS tropism of AAV-PHP.B and AAV-PHP.eB appears to extend to rats [14,15], whereas studies testing AAV-PHP.B or related capsids in nonhuman primates (NHPs) have yielded differing outcomes [16–18]. Surprisingly, the enhanced CNS tropism of AAV-PHP.B [6,9,12–15,19,20] is starkly absent in BALB/cJ mice [16]. These findings indicate that the ability of AAV-PHP.B to cross the BBB is affected by genetic factors that vary by species and mouse strain. In this work, we leverage this strain-dependence to identify LY6A as the cellular receptor responsible for the enhanced CNS tropism exhibited by the AAV-PHP.B capsid family, a finding consistent with a recent report from Hordeaux et al. (2019) [21]. In addition, we demonstrate that LY6A-mediated transduction can occur independently of known AAV9 receptors and is a unique means by which AAV-PHP.B capsids cross the mouse BBB. This has widespread implications for guiding the use of AAV-PHP.B capsids in disease models, as well as ongoing efforts to engineer next-generation AAVs that cross the BBB in other species.

## Results

### *Ly6* genetic variants associate with the CNS tropism of AAV-PHP.eB

**Fig 1:**
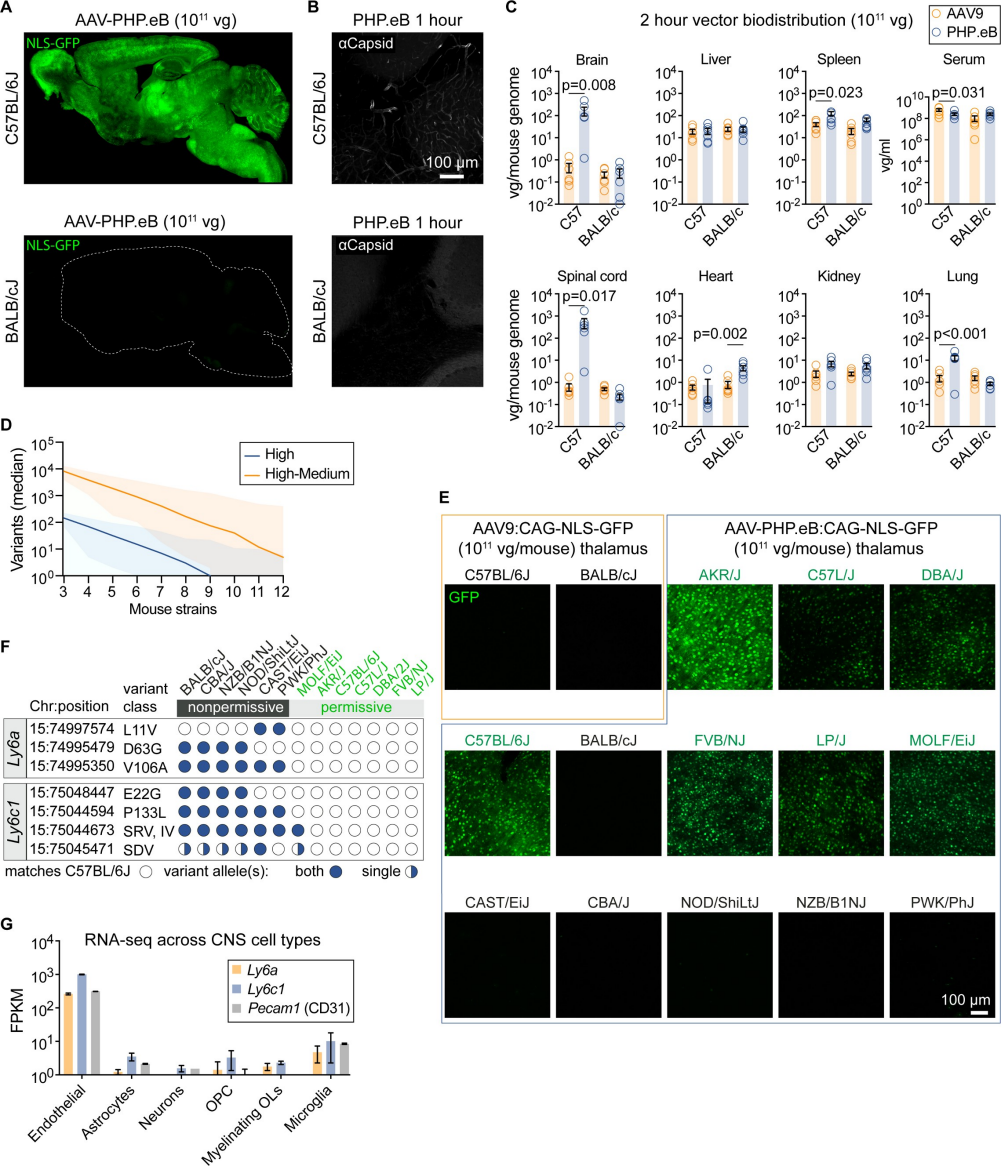
The nonpermissive AAV-PHP.eB CNS tropism phenotype associates with *Ly6* SNPs. (A) GFP within sagittal brain sections from C57BL/6J or BALB/cJ mice two weeks after intravenous administration of AAV-PHP.eB:CAG-NLS-GFP. (B) AAV capsid IHC within the cerebellum one hour after intravenous injection of AAV-PHP.eB. (C) Vector genome (vg) biodistribution of AAV-PHP.eB or AAV9 two hours after intravascular administration to C57BL/6J or BALB/cJ mice (n = 6/virus/line, mean ± s.e.m.; 2-way ANOVA). (D) The number of mouse lines predicted to be required to reduce the number of candidate variants associated with AAV-PHP.eB permissivity. The plotted lines depict the median number of simulated candidate variants; high (loss-of-function; blue) or high+medium (loss-of-function, missense, splicing variant; orange). Shaded regions represent 5–95th percentiles. (E) Native GFP fluorescence in the mouse thalamus two weeks after intravenous injection of 10^11^ vg/mouse CAG-NLS-GFP packaged into AAV9 (first two panels from top left) or AAV-PHP.eB. (F) *Ly6a* and *Ly6c1* SNPs correlate with the nonpermissive phenotype. Missense SNPs relative to C57BL/6J are listed as the amino acid change. SRV, splice region variant; IV, intron variant; SDV, splice donor variant. (G) Expression data (mean fragments per kilobase-million ± S.D.) for *Ly6a*, *Ly6c1*, and the endothelial cell marker, *Pecam1* [22].

The dramatically enhanced CNS tropism of AAV-PHP.B present in C57BL/6J but absent in BALB/cJ mice [16] extended to AAV-PHP.eB (Fig 1A) and two other AAV-PHP.B capsids, AAV-PHP.B2 and AAV-PHP.B3 (S1 Fig). Consistent with the reduced transduction phenotype in BALB/cJ mice, we observed a loss of the CNS-specific enhanced accumulation, relative to AAV9, of AAV-PHP.eB capsids (Fig 1B) and vector genomes (Fig 1C) along the vasculature and within the brains of BALB/cJ mice. These findings suggest that a cellular factor present on the brain endothelium is responsible for the efficient CNS-wide transduction mediated by the AAV-PHP.B capsids, and that this factor is absent or nonfunctional in BALB/cJ mice.

In light of this disparity, we sought to test for the enhanced CNS tropism of the AAV-PHP.B capsids across a panel of mouse lines, harnessing the natural genetic variation between mice to identify the genetic variants and, subsequently, candidate gene(s) responsible for the difference in CNS transduction by AAV-PHP.eB. Diverging from conventional diversity outbred or genetic linkage studies requiring the inter-breeding of mouse lines, we leveraged the open-source software Hail [23] to compile a database of genetic variants derived from whole-genome sequencing (WGS) data from the Mouse Genomes Project spanning 36 mouse lines [24]. Given the stark CNS transduction phenotype difference between strains, we hypothesized that susceptibility to AAV-PHP.eB is likely determined by a variation in a protein coding region of a single gene, and therefore aimed to rapidly identify variants whose alleles segregate with the observed phenotype across mice (permissive or non-permissive to enhanced CNS transduction by AAV-PHP.eB). Starting from millions of genetic variants comprised of single-nucleotide polymorphisms (SNPs) as well as insertions and deletions (indels), we narrowed our analysis to variants predicted to affect expression, splicing, or protein coding regions (for a list of variant types see S1 Table; the variant database is provided in S1 File). Using a statistical simulation framework, we estimated that 12 mouse lines would be sufficient to narrow our search to ~10 high/medium impact variants (Fig 1D, S1 Table), which could feasibly be experimentally interrogated for the enhanced AAV-PHP.eB CNS tropism. Based on this estimation, we acquired mice from 13 commercially available lines, including C57BL/6J and BALB/cJ, and administered 10^11^ vector genomes (vg)/animal of AAV-PHP.eB, which packaged an AAV genome encoding a green fluorescent protein (GFP) with a nuclear localization signal (NLS-GFP). Intravenous administration of AAV-PHP.eB resulted in robust GFP expression in the brains of permissive lines such as C57BL/6J, but not in those of nonpermissive mice such as BALB/cJ; we identified seven permissive and six nonpermissive lines (Fig 1E).

Using the permissivity data to filter the WGS dataset, we reduced the number of high and medium impact genetic variants to missense SNPs in the *Ly6a* and *Ly6c1* genes (Fig 1F). RNA sequencing data from sorted mouse brain cells (www.BrainRNAseq.org) [22] indicates that *Ly6a* and *Ly6c1* are expressed in brain endothelial cells (Fig 1G). Intriguingly, the mouse *Ly6* locus is linked to susceptibility to mouse adenovirus (MAV1) [25], which possesses an endothelial cell tropism that causes fatal hemorrhagic encephalomyelitis in C57BL/6 but not BALB/cJ mice [26]. The *LY6* gene family also influences susceptibility to infection by HIV1 [27,28], Flaviviridae (yellow fever virus, dengue, and West Nile virus [29]), Influenza A [30], and Marek’s disease virus in chickens [31]. Despite these promising links between *Ly6/LY6* genes and various virus susceptibilities, the mechanisms remain elusive and have not been leveraged in the development of gene therapy vectors.

**Fig 2:**
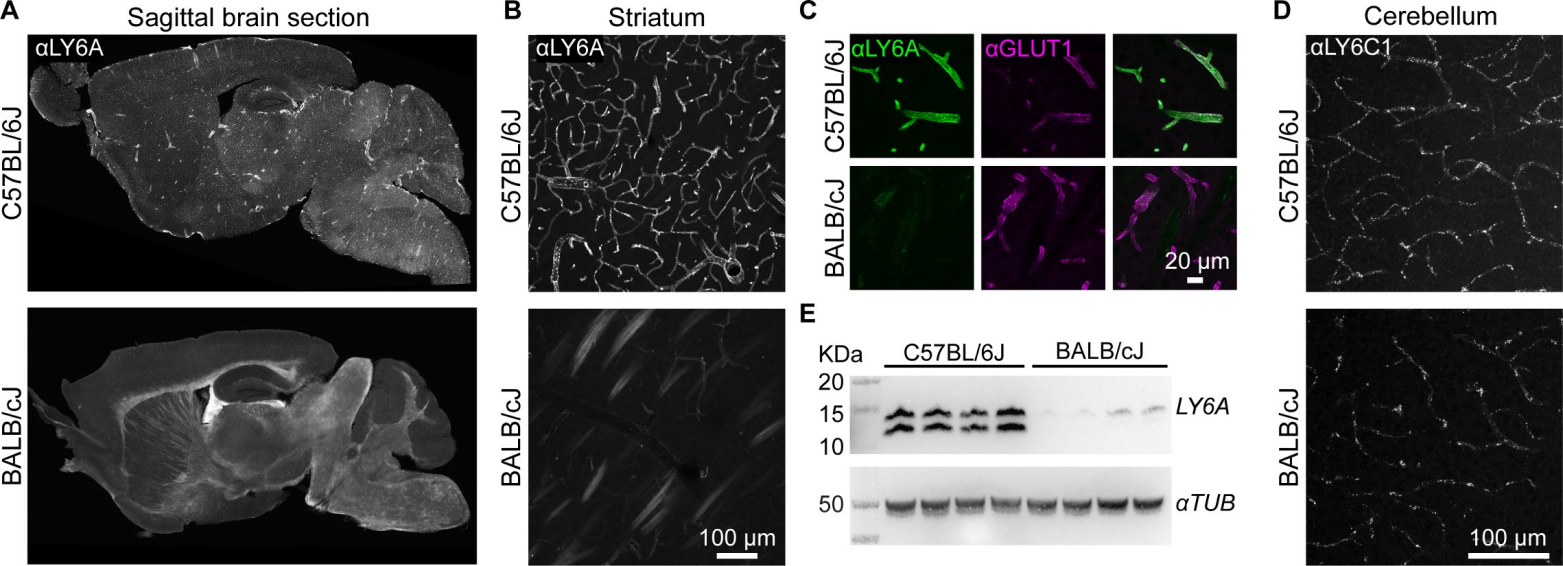
Brain *Ly6a* expression differs between C57BL/6J and BALB/cJ strains. (A) Representative images of LY6A IHC in sagittal brain sections from C57BL/6J or BALB/cJ mice. LY6A IHC in the striatum (B, white or C, green) with IHC for GLUT1 (magenta) to mark endothelial cells (see S2 Fig for images of LY6A IHC in brain sections from the 11 additional mouse lines). LY6C1 IHC in the cerebral cortex (D) of C57BL/6J or BALB/cJ mice. (E) Western blot from forebrain lysates reveals LY6A abundance and protein states in each mouse line (n = 4).

We first validated whether protein expression or localization differences arising from genetic variation within *Ly6a* or *Ly6c1* is associated with the differential AAV-PHP.eB tropism across mouse lines. Immunohistochemistry (IHC) assays revealed that LY6A is abundant within the CNS endothelium of C57Bl/6J mice but not in BALB/cJ mice (Fig 2A–2C). In contrast, *Ly6c1* was expressed on the CNS endothelium of both lines (Fig 2D). The reduced level of LY6A, relative to that observed in C57BL/6J mice, correlated with the nonpermissive AAV-PHP.eB transduction phenotype across five of six mouse strains (S2 Fig). This suggested that the reduced abundance of LY6A may contribute to, but is unlikely to be the only factor responsible for, the absence of enhanced AAV-PHP.eB transduction in nonpermissive strains. Indeed, by western blotting, the migration of bands recognized by a LY6A antibody differed in brain lysates from C57BL/6 and BALB/cJ mice. In C57BL/6J mice, two distinct bands are present, while in BALB/cJ mice only the upper band is detectable, suggesting that LY6A maturation or post-translational processing likely also contributes to the nonpermissive phenotype. We noted that the *Ly6a* V106A and *Ly6c1* P133L (Fig 1F) perfectly segregate with the nonpermissive phenotype (observed in six out of six nonpermissive mouse strains; absent in seven out of seven permissive mouse strains). Together, these results suggest an association between *Ly6* genetic variants and permissivity to transduction by AAV-PHP.eB.

### Ectopic *Ly6a* expression enhances transduction by AAV-PHP.eB in human cells

**Fig 3:**
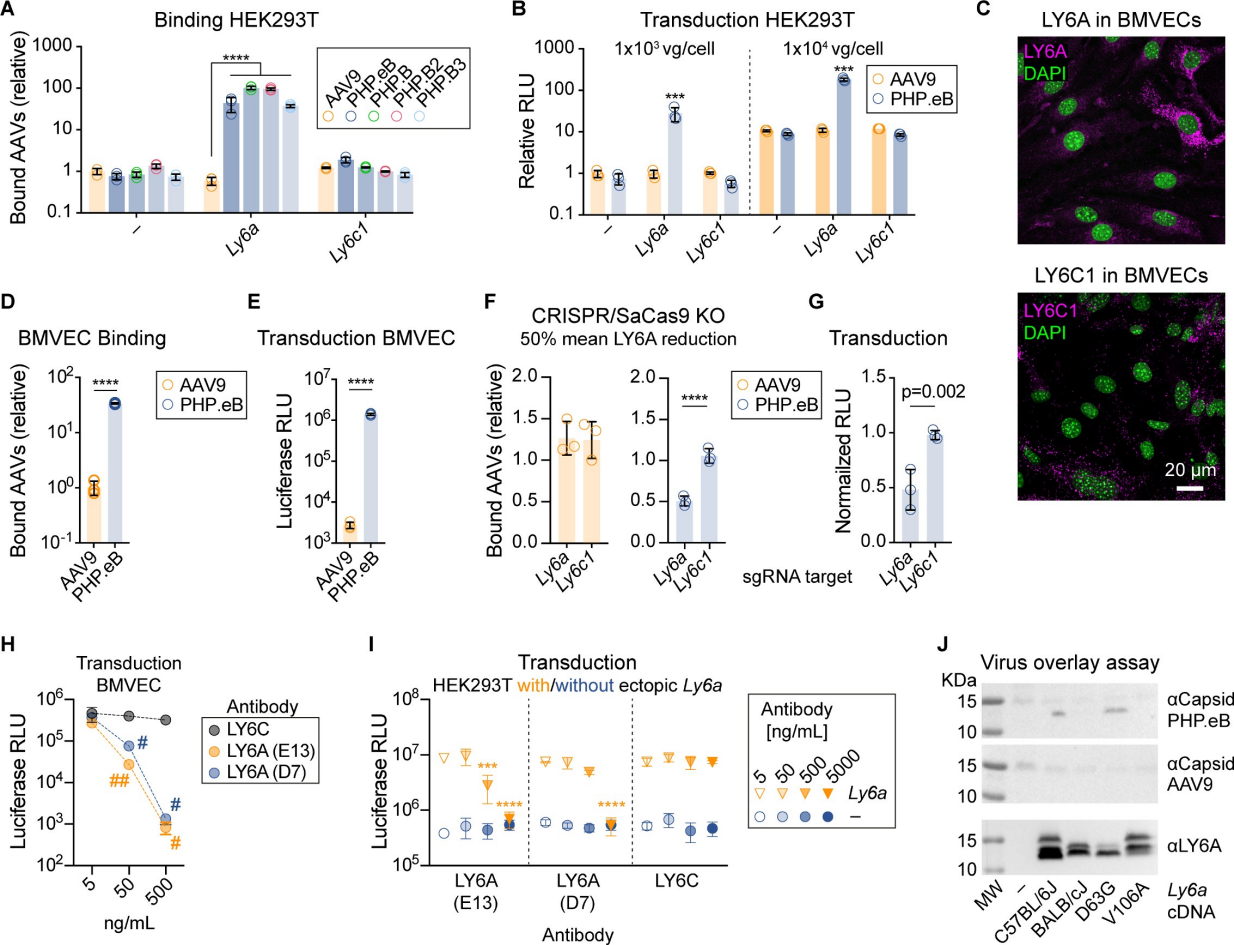
*Ly6a* mediates enhanced binding and transduction by AAV-PHP.eB. (A) Binding of the indicated virus to HEK293T cells transfected with *Ly6a* or *Ly6c1* or left untransfected (–). (B) Transduction measured by Luciferase assay normalized to AAV9 on mock transfected cells. (A, n = 3/group, ****p<0.0001; 2-way ANOVA, Tukey’s correction; B n = 3/group, ***p<0.001; 3-way ANOVA). (C) LY6A (magenta, top) and LY6C1 (magenta, bottom) immunostaining with nuclei (green, DAPI) in BMVECs. AAV9 and AAV-PHP.eB binding (D) and transduction (E) of BMVECs (n = 3/virus, mean ± S.D., ***p<0.0001; two-tailed t test). Binding was assessed by qPCR of the viral genome. Transduction was assessed by measuring Luciferase luminescence in relative light units (RLU). Binding (F; 2-way ANOVA, Dunnett’s multiple comparison test) and transduction (G; 1-way ANOVA, Sidak’s post test) by the indicated virus in cells treated with a vector containing an sgRNA to disrupt *Ly6a* or *Ly6c1* or no sgRNA (n = 3/group, mean ± S.D., ****p<0.0001). Data in F and G are normalized to cells treated with a control vector lacking a sgRNA sequence. Each data point represents cells that received a different sgRNA. (H-I) AAV-PHP.eB-mediated transduction (Luciferase RLU) of BMVECs (H) or HEK293 cells mock transfected (-) or transfected with *Ly6a* (I) following the pre-incubation of cells with the indicated antibody (n = 2/group, H; or n = 3/group, I, #p = 0.023, ##p = 0.010, ***p = 0.001, ****p<0.0001, αLY6C vs. αLY6A, 2-way ANOVA, Tukey’s correction for multiple comparisons). (J) Virus overlay assay using lysates from HEK293T cells transfected with *Ly6a* cDNAs from C57BL/6J or containing one or both BALB/cJ SNPs. Panels show immunoblotting for AAV capsid proteins after overlaying with AAV-PHP.eB or AAV9. Bottom panel shows the αLY6A signal from the same blot previously overlaid with AAV-PHP.eB.

*Ly6a* and *Ly6c1* are absent in primates [32]; therefore, we next asked whether ectopic *Ly6a* or *Ly6c1* expression in human cells would increase their susceptibility to AAV-PHP.eB. We transiently transfected HEK293T cells with cDNAs encoding C57BL/6J *Ly6a* or *Ly6c1*, and evaluated the effects on binding and transduction by AAV-PHP.B capsids. Remarkably, *Ly6a* expression resulted in a >50-fold increase in binding by each of the AAV-PHP.B capsids to HEK293T cells, but did not increase binding by AAV9 (Fig 3A). Expression of *Ly6a*, but not *Ly6c1*, also enhanced transduction by AAV-PHP.eB by 30-fold compared to the untransfected control (Fig 3B).

### Interaction with *Ly6a* is necessary for the enhanced transduction phenotype of AAV-PHP.eB

We next sought to determine whether LY6A is necessary for AAV-PHP.eB binding and transduction in CNS endothelial cells. To test this, we performed *Ly6a* and *Ly6c1* knockout experiments in primary brain microvascular endothelial cells (BMVECs) from C57BL/6J mice, which express both genes (Fig 3C) and are more efficiently bound and transduced by AAV-PHP.eB than by AAV9 (Fig 3D and 3E). We used CRISPR/SaCAS9 [33] and *Ly6a-* or *Ly6c1*-specific sgRNAs to disrupt each gene. Using three different sgRNAs to target *Ly6a*, we achieved a ~50% reduction of LY6A (S3 Fig) and a 50% reduction in binding by AAV-PHP.eB, but not AAV9 (Fig 3F and S3 Fig). A similar reduction in transduction by AAV-PHP.eB was observed (Fig 3G); AAV9 transduction of BMVECs was inefficient and not included for comparison. None of the sgRNAs targeting *Ly6c1* affected AAV-PHP.eB or AAV9 binding to the BMVECs (Fig 3F).

To confirm that the interaction between AAV-PHP.eB and LY6A is required for the enhanced transduction phenotype of AAV-PHP.eB, we preincubated BMVECs with LY6A-specific antibodies and then exposed the cells to AAV-PHP.eB. In a dose-dependent manner, either of two monoclonal LY6A-specific antibodies inhibited BMVEC transduction by AAV-PHP.eB, while a LY6C1-specific antibody had no effect (Fig 3H). The LY6A antibodies that blocked transduction of BMVECs had no impact on AAV-PHP.eB-mediated transduction of HEK293 cells (Fig 3I), but did block the increased transduction observed in HEK293 cells ectopically expressing *Ly6a*.

Collectively, the reduced AAV-PHP.eB binding resulting from *Ly6a* disruption in BMVECs, the complete inhibition of the enhanced transduction phenotype of AAV-PHP.eB in *Ly6a*-expressing cells by LY6A-specific antibodies, the high level of *Ly6a* expression within the CNS endothelium of permissive mouse lines, and the association of *Ly6a* SNPs with the nonpermissive phenotype support the conclusion that LY6A functions as a receptor for AAV-PHP.eB.

### Binding between LY6A and AAV-PHP.eB is disrupted by a V106A variant of LY6A

To determine whether AAV-PHP.eB directly binds LY6A and whether either of the missense SNPs (D63G or V106A) in the BALB/cJ *Ly6a* gene (Fig 1F) affect binding to AAV-PHP.eB, we performed virus overlay assays [34]. AAV-PHP.eB bound a protein that co-migrates with LY6A (Fig 3J) from HEK293T cells transfected with the C57BL/6J or D63G *Ly6a* cDNAs, but not from cells expressing *Ly6a* from the BALB/cJ or V106A cDNAs. Interestingly, the V106A variant is located near the predicted cleavage and GPI anchoring site (ω), and is predicted to reduce the likelihood of GPI-anchor modification [35] (S2 Table). The V106A SNP and the reduction of LY6A levels observed in five of six nonpermissive strains both likely contribute to the absence of a strong CNS tropism phenotype in these strains.

### LY6A enhances AAV-PHP.eB transduction independently of known AAV9 receptors

To determine whether LY6A acts solely as a primary attachment factor or has additional roles in promoting the internalization and trafficking of AAV-PHP.eB, we explored whether AAV-PHP.eB binding and transduction are dependent on known receptor interactions. AAVs typically use multiple cellular receptors for attachment and for internalization and intracellular trafficking [36]; AAV9 utilizes galactose as an attachment factor [37], and, like most AAVs, requires the AAV receptor (AAVR) for intracellular trafficking and transduction [34].

**Fig 4:**
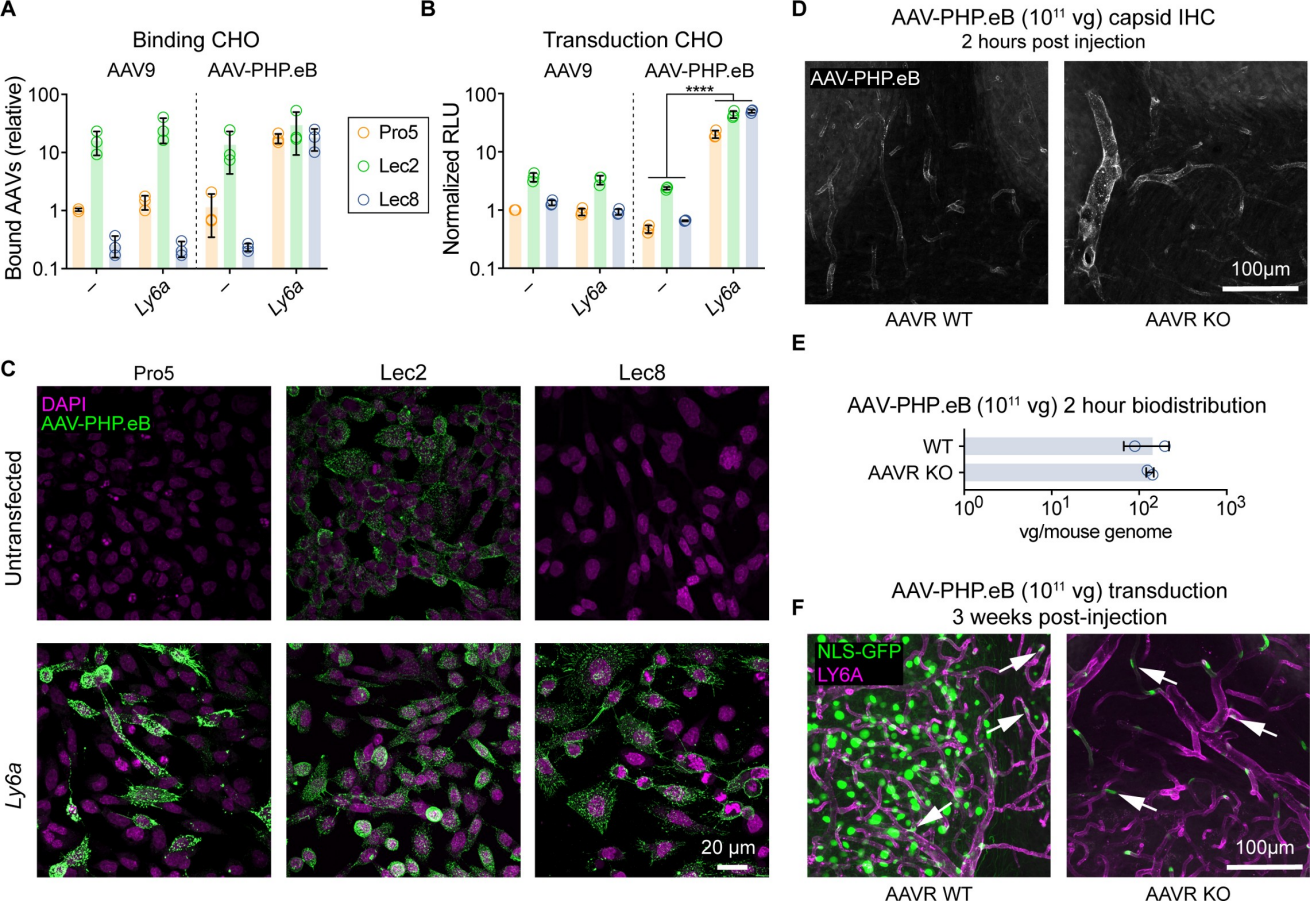
*Ly6a* expression renders cells permissive to AAV-PHP.eB transduction in the absence of galactose or AAVR. (A-C) AAV-PHP.eB or AAV9 viruses were added to control Pro5 CHO cells, Lec2 CHO cells with excess galactose, or Lec8 CHO cells deficient for galactose transfer. (A) Quantification of AAV binding to CHO cell derivatives via qPCR for viral genomes. (B) Transduction of CHO cells as measured by Luciferase assay 48 hours after virus addition, normalized to values from Pro5 cells transduced with AAV9. (C) AAV-PHP.eB capsid immunostaining of CHO cells that were untransfected (top row) or transfected with *Ly6a* (bottom row). (D-F) WT or AAVR KO mice were intravenously injected with AAV-PHP.eB:CAG-*NLS-GFP* (10^11^ vg/mouse) and brain tissue was assessed via IHC for capsid binding (D), vector genome biodistribution analysis (E) at two hours, or transduction (F) at three weeks post-injection (D-F: n = 2 per group/per experiment, mean ± SD). (F) Representative images of GFP fluorescence (green) with LY6A IHC (magenta).

We first tested the effect of varying levels of galactose on cell surface glycoproteins on LY6A-mediated AAV-PHP.eB binding. Using Pro5 Chinese Hamster ovary (CHO) cells and derivatives that expose excess galactose (Lec2 cells) or are unable to add galactose to their glycoproteins (Lec8 cells) [38], which were previously used to map the galactose binding site on the AAV9 capsid [37], we assessed whether AAV-PHP.eB-LY6A interactions are influenced by galactose levels. As expected, AAV9 and AAV-PHP.eB similarly bound and transduced Lec2 cells more efficiently than Lec8 or Pro5 cells (Fig 4A–4C), indicating that AAV-PHP.eB also utilizes galactose for cell attachment. In contrast, ectopic *Ly6a* expression selectively increased AAV-PHP.eB, but not AAV9, binding and transduction (Fig 4A and 4B) to Pro5 and Lec8 cells. *Ly6a* expression did not significantly increase binding of AAV-PHP.eB to Lec2 cells (Fig 4A), potentially due to the high levels of binding driven by excess galactose. Interestingly, *Ly6a* expression enhanced AAV-PHP.eB transduction of Pro5, Lec2, and Lec8 cells (Fig 4B). The finding that *Ly6a* expression rendered Lec8 cells as receptive to AAV-PHP.eB transduction as Pro5 cells indicates that LY6A functions as an attachment factor for AAV-PHP.eB independently of galactose. Furthermore, *Ly6a* expression enhanced AAV-PHP.eB transduction of Lec2 cells without increasing binding, suggesting that LY6A contributes to the internalization and/or trafficking of AAV-PHP.eB.

In this vein, we reasoned that this interaction may render *Ly6a*-expressing cells permissive to AAV-PHP.eB transduction in the absence of AAVR, which is essential for the intracellular trafficking of nearly all AAV capsids including AAV9 [39]. To test this possibility, WT and AAVR KO mice in the FVB/NJ genetic background (permissive to AAV-PHP.eB) [39] were injected with AAV-PHP.eB, and their brains were collected two hours later for capsid detection. We detected AAV-PHP.eB along the vasculature and in the brain lysates of both WT and AAVR KO mice (Fig 4D and 4E), indicating that AAV-PHP.eB binding to the brain vasculature occurs in the absence of AAVR. Three weeks post-administration, AAV-PHP.eB-mediated transduction of neurons and astrocytes, which do not express *Ly6a*, was nearly absent in the brains of AAVR KO mice (Fig 4F), suggesting that transduction of these cells remains dependent on AAVR. In contrast, AAV-PHP.eB-mediated transduction of *Ly6a*-expressing endothelial cells was evident throughout the brains of both WT and AAVR KO mice. These findings indicate that, by engaging LY6A, AAV-PHP.eB is capable of AAVR-independent binding and trafficking into CNS endothelial cells *in vivo*. Whether the interaction with LY6A is sufficient for AAV-PHP.eB transcytosis, or whether transcytosis remains dependent on AAVR is not yet clear. Collectively, these results demonstrate that AAV-PHP.eB uses a unique and unexpected endothelial cell receptor, LY6A, to gain access to the CNS.

## Discussion

The development of AAV-PHP.B capsids provided proof-of-concept that AAV vectors with dramatically enhanced BBB crossing capabilities can be engineered, without a priori mechanistic knowledge [5,6]. AAV-PHP.B and AAV-PHP.eB are now widely used vectors for mouse neuroscience studies. However, the species-specific tropism of the AAV-PHP.B capsids reduces their appeal for human CNS gene therapy and highlights the shortcomings of performing selections and screens in model systems–the enhanced features of the identified capsids may not extend beyond the context (in this case, the genetic background) in which the selective pressure was applied. Accordingly, as compared to efforts in mice, selections in nonhuman primates (NHPs) are predicted to result in the identification of capsids whose enhanced features better translate to humans. Nonetheless, such efforts to develop clinically relevant vectors may likewise be thwarted by the identification of species- or model-specific capsids. Therefore, the pursuit of a vector that crosses the human BBB with AAV-PHP.eB-like efficiency gains will be aided by a mechanistic understanding of how naturally isolated and engineered capsids cross the BBB.

In this study, we rapidly identified a single missense variant in *Ly6a*, out of a starting pool of millions of genetic variants, which segregates with efficient CNS transduction by AAV-PHP.eB. We accomplished this by first narrowing down candidates to genetic variants with a predicted high or medium impact and eliminating the bulk of the variants that did not segregate with the permissivity phenotype. This segregation study was achieved by leveraging Hail [23], the Mouse Genomes Project dataset [24], and 13 commercially available mouse lines; the code was implemented and run end-to-end on WGS data within hours, harnessing Hail’s ability to scale computation across a large compute cluster, and the *in vivo* screening was completed in three weeks. The speed and small number of animals required for this approach is unprecedented compared to the conventional approaches of using diversity outbred lines or breeding generations of mice to determine the approximate genomic loci that segregates with a given phenotype.

After narrowing down the perfectly segregating genetic variants to two missense SNPs in two genes, we used molecular and biochemical studies to identify and validate *Ly6a* as the gene encoding the receptor for the AAV-PHP.B capsids. Because we restricted this approach to high and medium impact variants, our study does not rule out the possibility that other perfectly segregating noncoding variants present within *Ly6a* or other sites within the genome may contribute to the CNS transduction phenotype. In addition, we are not able to rule out the possibility that other genetic variants present in a subset of the nonpermissive strains within and surrounding *Ly6a* (S4 Fig) contribute to the nonpermissive phenotype. It is possible that one or more of these variants may influence *Ly6a* expression and contribute to the variation in LY6A levels and localization observed across nonpermissive strains (S2 Fig).

Our finding that *Ly6a* expression increases binding by the top three AAV-PHP.B variants, harboring unique peptide insertions (TLAVPFK, SVSKPFL, and FTLTTPK), identified using CREATE [6] suggests that LY6A has properties that make it an ideal receptor to engage for efficient transcytosis across the C57BL/6J BBB. Indeed, LY6A facilitates binding and transduction by AAV-PHP.eB in cells lacking either of the known AAV9 receptors, galactose and AAVR. Furthermore, ectopic expression of *Ly6a* is sufficient to render both human and hamster cells permissive to the enhanced binding and transduction of AAV-PHP.eB. Importantly, these findings demonstrate that AAVs can be engineered to utilize entirely new cell entry/transduction mechanisms rendering the novel capsids less dependent on interactions with the receptors that natural AAV serotypes rely on for transduction.

Although there is no direct *Ly6a* homolog in primates, other cellular factors that share key properties with LY6A such as abundant luminal surface exposure on brain endothelium, localization within lipid micro-domains through GPI anchoring, or specific recycling/intracellular trafficking capabilities, may be prime molecular targets for gene delivery vectors in mice, NHPs, and humans. Notably, other LY6 proteins with homologs in primates are present within the CNS endothelium and can be explored and potentially harnessed for AAV capsid engineering. Developing capsids and/or other biologicals that target these receptors can open up new therapeutic avenues for treating a wide range of currently intractable neurological diseases.

## Materials and methods

### Mouse strain permutation analysis

Access to WGS data for 36 commercially available mouse lines [24] made it possible to estimate the number of lines necessary to produce a shortlist of candidate variants. Starting from the known permissive C57BL/6J and nonpermissive BALB/cJ strains, we used the genomic analysis software Hail [23] to simulate permissivity phenotypes for other mouse lines and compute the number of candidate variants in both the high and high-and-medium predicted functional impact classes. First, we sampled the probability *p* that a random mouse line is permissive from a *Beta(ɑ = 2*, *β = 2)* distribution, based on the two known mouse phenotypes. Second, we sampled a subset of additional commercially available lines (S3 Table). Third, we simulated a permissivity phenotype for each line in the subset by flipping a *p*-coin. Finally, we calculated the number of variants with perfect allelic segregation between the permissive and nonpermissive lines. We ran 500 iterations of this model for each of ten subset sizes (3 to 12 mice), providing a distribution over the number of candidate variants at each mouse sample size. This simulation informed our decision regarding the number of mouse lines to order and test in parallel.

### Plasmids and primers

The AAV-PHP.eB Rep-Cap trans plasmid was generated by gene synthesis (GenScript). AAV9, AAV-PHP.B, AAV-PHP.B2, and AAV-PHP.B3 were generated by replacing the AAV-PHP.eB variant region with that of AAV9, AAV-PHP.B, B2, or B3 using isothermal HiFi DNA Assembly (NEB). The reporter and *Ly6* expression vectors were cloned into an AAV-CAG-WPRE-hGH pA backbone obtained from Viviana Gradinaru through Addgene (#99122). GFP, 2A-luciferase, *Ly6a*, and *Ly6c1* (splice variant 1) cDNAs were synthesized as gBlocks (IDT). The NLS-GFP were synthesized using the N-terminal SV40 NLS sequence present in the Addgene plasmid #99130. The CMV-SaCAS9 vector (AAV-CMV::NLS-SaCas9-NLS-3xHA-bGHpA;U6::BsaI-sgRNA) was obtained from Dr. Feng Zhang through Addgene (#61591). sgRNAs specifically targeting *Ly6a* or *Ly6c1* were cloned after the U6 promoter using a single bridge oligo for each reaction as recommended (HiFi DNA Assembly, NEB). The Broad GPP sgRNA tool for SaCAS9 was used to identify suitable SaCAS9 target sites [40].

The following primers were used for *Ly6a* sgRNA cloning:

5’-CTTGTGGAAAGGACGAAACACCG**AATTACCTGCCCCTACCCTGA**GTTTTAGTACTCTGGAAACAG,

5’-CTTGTGGAAAGGACGAAACACCG**CTTTCAATATTAGGAGGGCAG**GTTTTAGTACTCTGGAAACAG,

5’-CTTGTGGAAAGGACGAAACACCG**AATATTGAAAGTATGGAGATC**GTTTTAGTACTCTGGAAACAG

The following primers were used for *Ly6c1* sgRNA cloning:

5’-CTTGTGGAAAGGACGAAACACCG**ACTGCAGTGCTACGAGTGCTA**GTTTTAGTACTCTGGAAACAG,

5’-CTTGTGGAAAGGACGAAACACCG**CAGTTACCTGCCGCGCCTCTG**GTTTTAGTACTCTGGAAACAG,

5’-CTTGTGGAAAGGACGAAACACCG**GATTCTGCATTGCTCAAAACA**GTTTTAGTACTCTGGAAACAG

The following primers were used for biodistribution assays and/or binding assays

GFP: 5’-TACCCCGACCACATGAAGCAG, 5’-CTTGTAGTTGCCGTCGTCCTTG

Mouse *Gcg*: 5’-AAGGGACCTTTACCAGTGATGTG, 5’-ACTTACTCTCGCCTTCCTCGG

Human *GCG*: 5’-ATGCTGAAGGGACCTTTACCAG, 5’-ACTTACTCTCGCCTTCCTCGG

CHO *Gcg*: 5’-ATGCTGAAGGGACCTTTACCAG, 5’-CTCGCCTTCCTCTGCCTTT

### CRISPR/SaCas9 KO experiments

AAV-PHP.eB vectors with sgRNA sequences targeting *Ly6a* and *Ly6c1* were generated and purified to knockout the respective genes in C57BL/6 mouse primary brain microvascular endothelial cells (CellBiologics, Cat. #C57-6023). AAV vectors (1x10^6^ vg/cell) were used to transduce cells every three days for three rounds to achieve higher knockout efficiency. Cells were passaged as necessary.

### Cell lines and primary cultures

HEK293T (HEK293T/17; CRL-11268), Pro5 (CRL-1781), Lec2 (CRL-1736), and Lec8 (CRL-1737) were obtained from ATCC. BMVECs were obtained from Cell Biologics (C57-6023) and cultured as directed by the manufacturer.

### Virus production and purification

Recombinant AAVs were generated by triple transfection of HEK293T cells using polyethylenimine (PEI) and purified by ultracentrifugation over iodixanol gradients as previously described [6].

### Western blotting and virus overlay assays

The virus overlay assay was performed as previously reported [34] with some modifications. Briefly, protein lysates were separated on Bolt 4–12% Bis-Tris Plus gels and transferred onto nitrocellulose membranes. After incubation with AAV9 or PHP.eB at 5e11 vg/mL, the membranes were fixed with 4% PFA at room temperature for 20 minutes to crosslink the interaction between the capsid and its target protein, followed by 2M HCl treatment at 37°C for 7 minutes to expose the internal capsid epitope for detection. The blots were then rinsed and incubated with anti-AAV VP1/VP2/VP3 (1:20; American research products, Inc., cat. #03–65158), anti-LY6A (1:1000; BD, 553333 or 557403) or anti-alpha-tubulin (1:1000; Sigma, T9026) followed by incubation with a horseradish peroxidase (HRP)-conjugated secondary antibody at 1:5000. The detection of the HRP signal was by SuperSignal West Femto Maximum Sensitivity Substrate using a Bio-Rad ChemiDoc ^TM^ MP system #1708280.

### Animals

All procedures were performed as approved by the Broad Institute IACUC (0213-06-18) or Massachusetts General Hospital IACUC (2014N000005; AAVR experiments). AKR/J (000648), BALB/cJ (000651), CBA/J (000656), CAST/EiJ (000928), C57Bl/6J (000664), C57BL/J (000668), DBA/2J (000671), FVB/NJ (001800), LP/J (000676), MOLF/EiJ (000550), NOD/ShiLtJ (001976), NZB/B1NJ (000684), and PWK/PhJ (003715) were obtained from the Jackson Laboratory (JAX). AAVR KO mice were a generous gift from Dr. J.E. Carette (Stanford) to Dr. Balazs and have been previously described [39]. Recombinant AAV vectors were administered intravenously via the retro-orbital sinus in young adult male or female mice. Mice were randomly assigned to groups based on predetermined sample sizes. No mice were excluded from the analyses. Experimenters were not blinded to sample groups.

### Tissue processing, immunohistochemistry, and imaging

Mice were anesthetized with Euthasol (Broad) or ketamine (MGH) and transcardially perfused with phosphate buffered saline (PBS) at room temperature followed by 4% paraformaldehyde (PFA) in PBS. Tissues were post-fixed overnight in 4% PFA in PBS and sectioned by vibratome. IHC was performed on floating sections with antibodies diluted in PBS containing 10% donkey serum, 0.1% Triton X-100, and 0.05% sodium azide. Primary antibodies were incubated at room temperature overnight. The sections were then washed and stained with secondary (Alexa-conjugated antibodies, 1:1000) for four hours or overnight. Primary antibodies used were mouse anti-AAV capsid (1:20; American Research Products, 03–65158, clone B1), LY6A (1:250; BD Bioscience, E13: 553333 or D7: 557403), LY6C (1:250; Millipore-Sigma, MABN668), Glut1 (1:250; Millipore Sigma, 07–1401). To expose the internal B1 capsid epitope in intact capsids, tissue sections or cells on coverslips were treated for 15 or 7 minutes, respectively, with 2M HCl at 37°C. The treated samples were then washed extensively with PBS prior to addition of the primary antibody.

### *In vivo* vector and capsid biodistribution

Five- to six-week-old C57Bl/6J mice, BALB/cJ mice, AAVR WT FVB/NJ, or AAVR KO FVB/NJ mice were injected intravenously with 10^11^ vg of AAV vector packaged into the indicated capsid. One or two hours after injection, the mice were perfused with PBS and tissues were collected and frozen at -80°C. Samples were processed for AAV genome biodistribution analysis by measuring the number of vector genomes (vg) in the sample using absolute qPCR for the GFP sequence. The values are reported as vg/mouse genome after normalizing to the number of copies of host cell genomes using primers recognizing mouse *Gcg* or human *GCG* as previously described [6]. To visualize the capsid distribution, mice were perfused with 4% PFA after dosing with AAV vector, and brains were sectioned into 100 μM and labeled with the indicated antibodies. Perfusions were performed after sodium pentobarbital euthanasia. All efforts were made to minimize suffering.

### Microscopy

Images were taken on an Axio Imager.Z2 Basis Zeiss 880 laser scanning confocal microscope fitted with the following objectives: PApo 10x/0.45 M27, Plan-Apochromat 20x/0.8 M27, or Plan-APO 40x/1.4 oil DIC (UV) VIS-IR, or on a Zeiss Imager.M2 (5x tiled, whole sagittal section images). In some whole sagittal section images, the 16-bit green channel (GFP) gamma was adjusted to enable visualization of low- and high-expressing cells while minimizing over-saturation. In all cases, changes to contrast or gamma as well as microscope laser settings were kept constant across sets of images that were directly compared.

### *In vitro* bound vector genome assays

The cDNA of *Ly6* family members (0.5 μg/well) were transfected into HEK293T cells (3x10^5^/well) using PEI or into CHO cells (1.5x10^5^/well) with lipofectamine 3000 reagent (ThermoFisher, L3000001) in 24-well plates. 48 hours later, the cells were chilled to 4°C and the media was exchanged with fresh cold media containing the indicated recombinant AAV (10^5^ vg/cell). One hour later, cells were washed three times with cold PBS, then fixed with 4% PFA for IHC or lysed for genomic DNA extraction and qPCR analyses. Vector genomes bound were normalized to the number of cell genomes by using primers specific to *Gcg or GCG*. For BMVECs, 2x10^4^ cells/well were seeded in 12-well plates the day before exposure to virus. The assay was performed as above except that the AAV vectors were added at 10^6^ vg/cell.

### Luciferase transduction assay

*Ly6* family members (0.1 μg/well) were transfected into the indicated cells (HEK293/17: 4x10^5^/well; CHO: 2.5x10^4^/well, BMVECs: 5x10^3^/well) in 96-well plates (PerkinElmer, 6005680) in triplicate. 48 hours later, cells were transduced with AAV-CAG-GFP-2A-Luciferase-WPRE packaged into AAV9 or AAV-PHP.eB. Luciferase assays were performed with Britelite plus Reporter Gene Assay System (PerkinElmer, 6066766). Luciferase activity was reported as relative light units (RLU) as raw data or normalized to non-transfected control wells transduced with AAV9, or a control transduced without a sgRNA (Fig 3F).

### Antibody inhibition assays

BMVECs were seeded at 2,000 cells/well in a 96-well plate. The following day, cells were incubated with LY6C or LY6A rat IgG antibodies at indicated concentration (5, 50, or 500 ng/mL) at 4°C with gentle shaking for one hour. AAV-PHP.eB:CAG-GFP-2A-Luc vectors were added at 13,000 vg/cell at 37°C and the Luciferase reporter assay was performed 48 hours post-transduction. Antibody inhibition assays were performed on HEK293 cells as above with the following changes: HEK293 cells were seeded at 20,000 cells/well in 96-well plate the day before transfection with *Ly6a* or control DNA (pUC19). The cells were incubated with AAV-PHP.eB:CAG-GFP-2A-Luc vectors (1,300 vg/cell) at 37°C.

### Statistical analysis and experimental design

Microsoft Excel and Prism 8 were used for data analysis. For the comparison between AAV9 and AAV-PHP.eB biodistribution, a group size of six per group (three males and three females) was used based on prior data that indicated a large effect size. No animals or samples were excluded from the analysis. S1 Fig images are representative of two animals per group. To evaluate AAV-PHP.eB in the 13 mouse lines, AAV9 (n = 1, 10^11^ vg/animal) or AAV-PHP.eB (n = 2; one per dose at 10^11^ and 10^12^ vg/animal). LY6A IHC in S2 Fig is representative of two animals per line. *In vitro* transduction and binding experiments display the means from three independent experiments. In Fig 3F and 3G, each data point represents a different sgRNA, each averaged from three independent experiments. Data were normalized separately for each AAV capsid to cells transduced with SaCas9 vectors without an sgRNA. S3 Fig presents the same data as Fig 3F separated by each individual sgRNA. Data from AAVR WT and KO mice are representative of two mice per genotype per time point post-injection. In all panels, ***p<0.001 and ****p<0.0001, p values >0.001 are provided in the figures as exact values. In all experiments, n refers to distinct animals for *in vivo* assays or distinct samples or transfections in cell culture assays.

### Data and code availability

The compiled variant dataset, filtered for variants of predicted high and medium impact, from all mice within the Mouse Genomes Project is provided as a resource to the research community (S1 File). The code used to generate the variant dataset and for the permutation analysis are deposited to a GitHub repository available at https://github.com/tpoterba/mouse-PHP.eB-simulation.

## Supporting information

S1 Table The types of genetic variants included in the Mouse Genomes Project WGS analysis. The predicted variant types, their count among all 36 mouse strains in the mouse genome project [1,2] database, and their predicted impact is shown. We restricted the analysis to variants with medium or high predicted impact on gene expression or coding sequence.
(PDF)

S2 Table LY6A from C57Bl/6J but not BALB/cJ mice is predicted to be GPI anchored. The predGPI prediction model [3] was used to assess the probability that the alleles of *Ly6a* present in permissive and nonpermissive mouse strains encode protein products likely to be modified by a GPI anchor. Within the amino acid sequences, SNPs are highlighted in red and underlined text; predicted GPI-anchor sites (ω-sites) are highlighted in bold green text, and cleaved C-terminal hydrophobic tail sequences are highlighted in magenta text.
(PDF)

S3 Table Permissive or nonpermissive AAV-PHP.eB CNS transduction phenotypes for inbred mouse lines with available WGS data. The lines highlighted in bolded text were used in the analysis presented in Fig 1D.
(PDF)

S1 Fig Transduction of the brains of BALB/cJ and C57BL/6J mice by AAV9, AAV-PHP.B, AAV-PHP.eB, AAV-PHP.B2, or AAV-PHP.B3. Images of GFP fluorescence in whole brain sagittal sections from C57BL/6J (left column) or BALB/cJ (right column) two weeks after intravenous injection of 1x10^11^ vg/mouse AAV-CAG-NLS-GFP packaged into the indicated capsid.
(TIF)

S2 Fig LY6A is highly abundant on the brain endothelium of permissive mouse lines, but its distribution and/or expression is altered in mice nonpermissive to AAV-PHP.eB CNS transduction. Sagittal whole brain images show LY6A IHC in several representative permissive and nonpermissive mouse lines.
(TIF)

S3 Fig Disruption of Ly6a and Ly6c1 using CRISPR/Cas9 and target-specific sgRNAs resulted in reduced LY6A protein and reduced AAV-PHP.eB binding. (A) The individual sgRNA data used to generate Fig 3D. (B) Western blots for LY6A (top) or TUBULIN (bottom) in lysates prepared from BMVECs treated with the individual sgRNAs shown in (A). (C) LY6A Western blot band intensity quantification by densitometry.
(TIF)

S4 Fig Genetic variants within the region surrounding the *Ly6a* gene. **The schematic on the left shows the structure of the exons and introns of the *Ly6a* isoform 1.** The genetic variants present in at least one of the nonpermissive strains (column headers in blue text) but not the permissive strains (column headers in green) are shown. Variants that segregate between permissive and nonpermissive strains are highlighted in orange text. Variants within exons are highlighted in gray. Variants are shown as homozygous (2), heterozygous (1), absent (0), or as data not available (-).
(PDF)

S1 File The S1_File.csv is a comma-separated values table of the high and medium impact variants present in the 35 Mouse Genomes Project mouse strains relative to the C57BL/6J reference assembly. The presence of the gene variants is given as 1 for heterozygous, 2 for homozygous, or 0 for absent.
(ZIP)

S2 File References cited in the supplementary information. (PDF)

S3 File Uncropped blot images. (PDF)

S4 File Data points used to generate all figure plots. (XLSX)
