# Supplementary material for: Delivering genes across the blood-brain barrier: LY6A, a novel cellular receptor for AAV-PHP.B capsids
Source: PLoS One. 2019 Nov 14;14(11):e0225206. doi: 10.1371/journal.pone.0225206 (PMC6855452; doi:10.1371/journal.pone.0225206)
Supplement: S1 Table — The predicted variant types, their count among all 36 mouse strains in the mouse genome project [1,2] database, and their predicted impact is shown. We restricted the analysis to variants with medium or high predicted impact on gene expression or coding sequence. (PDF) [file pone.0225206.s001.pdf]

S1 Table. The types of genetic variants included in the Mouse Genomes Project WGS analysis.

Abbreviation	Variant type	Count	Impact
INT	Intron variant	38745814	Low
DWNGV	Downstream genetic variant	9820625	Low
UPGV	Upstream genetic variant	9709435	Low
NTV	Noncoding transcript variant	5864559	Low
NTEV	Noncoding transcript exon variant	1041185	Low
3UTR	3' Prime UTR variant	823815	Low
SYN	Synonymous variant	344244	Low
MS	Missense variant	216611	Medium
5UTR	5' UTR variant	128375	Low
NMD	NMD transcript variant	123148	Low
SRV	Splice region variant	80431	Medium
INFD	Inframe deletion	3502	Medium
FST	Frameshift variant	2711	High
SDV	Splice donor variant	2609	HIgh
INFI	Inframe insertion	2452	Medium
SG	Stop gained	2450	HIgh
SAV	Splice acceptor variant	1922	High
MIR	Mature miRNA variant	1220	Low
STPRV	Stop retained variant	370	Low
STPL	Stop lost	332	High
SRTL	Start lost	322	Medium
CSV	Coding sequence variant	193	Low
PAV	Protein altering variant	87	Low
ITCV	Incomplete terminal codon variant	75	Low
STPRV	Start retained variant	31	Low

The predicted variant types, their count among all 36 mouse strains in the mouse genome project [1,2] database, and their predicted impact is shown. We restricted the analysis to variants with medium or high predicted impact on gene expression or coding sequence.
