# Supplementary material for: Delivering genes across the blood-brain barrier: LY6A, a novel cellular receptor for AAV-PHP.B capsids
Source: PLoS One. 2019 Nov 14;14(11):e0225206. doi: 10.1371/journal.pone.0225206 (PMC6855452; doi:10.1371/journal.pone.0225206)
Supplement: S2 Table — The predGPI prediction model [3] was used to assess the probability that the alleles of Ly6a present in permissive and nonpermissive mouse strains encode protein products likely to be modified by a GPI anchor. Within the amino acid sequences, SNPs are highlighted in red and underlined text; predicted GPI-anchor sites (ω-sites) are highlighted in bold green text, and cleaved C-terminal hydrophobic tail sequences are highlighted in magenta text. (PDF) [file pone.0225206.s002.pdf]

S2 Table. LY6A from C57Bl/6J but not BALB/cJ mice is predicted to be GPI anchored.

Gene (strain)	ω-site prediction	Specificity	Probability	Sequence
Ly6a (C57Bl/6J;DBA/J; AKR/J)	110	100%	Highly Probable	MDTSHTTKSCLLILLVALLCAERAQGLECYQCYGVPFETSCPSITCP<br>YPDGVCVTQEAAVIVDSQTRKVKNNLCLPICPPNIESMEILGTKVNV<br>KTSCCQEDLCNVAVP**N**GGSTWTMAGVLLFSLSSVLLQTLL
Ly6a (CAST/EiJ; PWK/PhJ)	110	0%	Not GPI-anchored	MDTSHTTKSC**V**LILLVALLCAERAQGLECYQCYGVPFETSCPSITC<br>PYPDGVCVTQEAAVIVDSQTRKVKNNLCLPICPPNIESMEILGTKVN<br>VKTSCCQEDLCN**A**AVP**N**GGSTWTMAGVLLFSLSSVLLQTLL
Ly6a (BALB/C;NOD/ShiLtJ)	110	0%	Not GPI-anchored	MDTSHTTKSCLLILLVALLCAERAQGLECYQCYGVPFETSCPSITCP<br>YPDGVCVTQEAAVIV**G**SQTRKVKNNLCLPICPPNIESMEILGTKVNV<br>KTSCCQEDLCN**A**AVP**N**GGSTWTMAGVLLFSLSSVLLQTLL
Ly6c1 (C57Bl/6J)	102	100%	Highly Probable	MDTSHTTKSCVLILLVALLCAERAQGLQCYECYGVPIETSCPAVTC<br>RASDGFCIAQNIELIEDSQRRKLKTRQCLSFCPAGVPIRDPNIRERT<br>SCCSEDLC**N**AAVPTAGSTWTMAGVLLFSLSSVVLQTLL
Ly6e	107	100%	Highly Probable	MSATSNMRVFLPVLLAALLGMEQVHSLMCFSCTDQKNNINCLWPV<br>SCQEKDHYCITLSAAAGFGNVNLGYTLNKGCSPICPSENVNLNLGV<br>ASVNSYCCQSSFCNF**S**AAGLGLRASIPLLGLGLLLSLLALLQLSP

The predGPI prediction model [3] was used to assess the probability that the alleles of *Ly6a* present in permissive and nonpermissive mouse strains encode protein products likely to be modified by a GPI anchor. Within the amino acid sequences, SNPs are highlighted in red and underlined text; predicted GPI-anchor sites (ω-sites) are highlighted in bold green text, and cleaved C-terminal hydrophobic tail sequences are highlighted in magenta text.
