# Supplementary material for: Delivering genes across the blood-brain barrier: LY6A, a novel cellular receptor for AAV-PHP.B capsids
Source: PLoS One. 2019 Nov 14;14(11):e0225206. doi: 10.1371/journal.pone.0225206 (PMC6855452; doi:10.1371/journal.pone.0225206)
Supplement: S3 Table — The lines highlighted in bolded text were used in the analysis presented in Fig 1D. (PDF) [file pone.0225206.s003.pdf]

S3 Table. Permissive or nonpermissive AAV-PHP.eB CNS transduction phenotypes for inbred mouse lines with available WGS data.

Strain	Enhanced CNS tropism	References
**AKR/J**	Present	This study
**BTBR/T+/Itpr3tf/J**	Present	Predicted
**C57BL/6J**	Present, previously reported	[4–7]
**C57BL/6NJ**	Present, previously reported	[8]
**C57BL/10J**	Present	Predicted
**C57BR/cdJ**	Present	Predicted
**C57L/J**	Present	This study
C58/J	Present	Predicted
**DBA/1J**	Present	Predicted
**DBA/2J**	Present	This study
**FVB/NJ**	Present	This study
I/LnJ	Present	Predicted
KK/HiJ	Present	Predicted
**LP/J**	Present	This study
**NZW/LacJ**	Present	Predicted
RF/J	Present	Predicted
**MOLF/EiJ**	Present	This study
129P2/OlaHsd	Present	Predicted
**129S1/SvImJ**	Present	Predicted
129S5SvEvBrd	Present	Predicted
**A/J**	Absent	Predicted
**BALB/cJ**	Absent, previously reported	[9]; this study
BUB/BnJ	Absent	Predicted
**CAST/EiJ**	Absent	This study
**CBA/J**	Absent	This study
C3H/HeH	Absent	Predicted
**C3H/HeJ**	Absent	Predicted
**LEWES/EiJ**	Absent	Predicted
**NOD/ShiLtJ**	Absent	This study
**NZB/B1NJ**	Absent	This study
**NZO/HILtJ**	Absent	Predicted
**PWK/PhJ**	Absent	This study
SEA/GnJ	Absent	Predicted
**SPRET/EiJ**	Absent	Predicted
SEA/GnJ	Absent	Predicted
ST/bJ	Absent	Predicted
**WSB/EiJ**	Absent	Predicted
ZALENDE/EiJ	Absent	Predicted

The lines highlighted in bolded text were used in the analysis presented in Fig 1D.
