# Supplementary figures and images for: Delivering genes across the blood-brain barrier: LY6A, a novel cellular receptor for AAV-PHP.B capsids
Source: PLoS One. 2019 Nov 14;14(11):e0225206. doi: 10.1371/journal.pone.0225206 (PMC6855452; doi:10.1371/journal.pone.0225206)

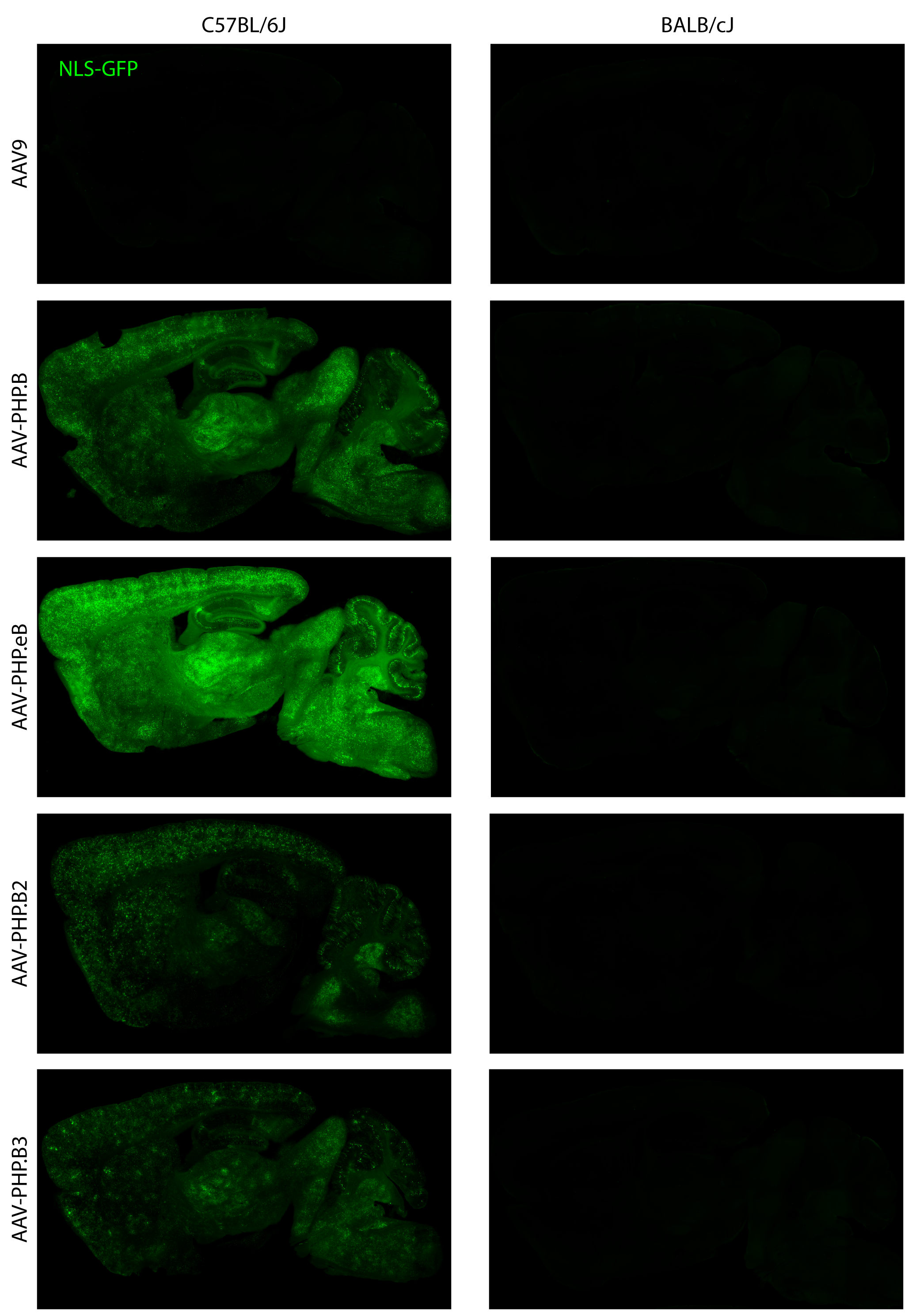

Supplement: S1 Fig — Images of GFP fluorescence in whole brain sagittal sections from C57BL/6J (left column) or BALB/cJ (right column) two weeks after intravenous injection of 1x10^11^ vg/mouse AAV-CAG-NLS-GFP packaged into the indicated capsid. (TIF) [file pone.0225206.s004.tif]

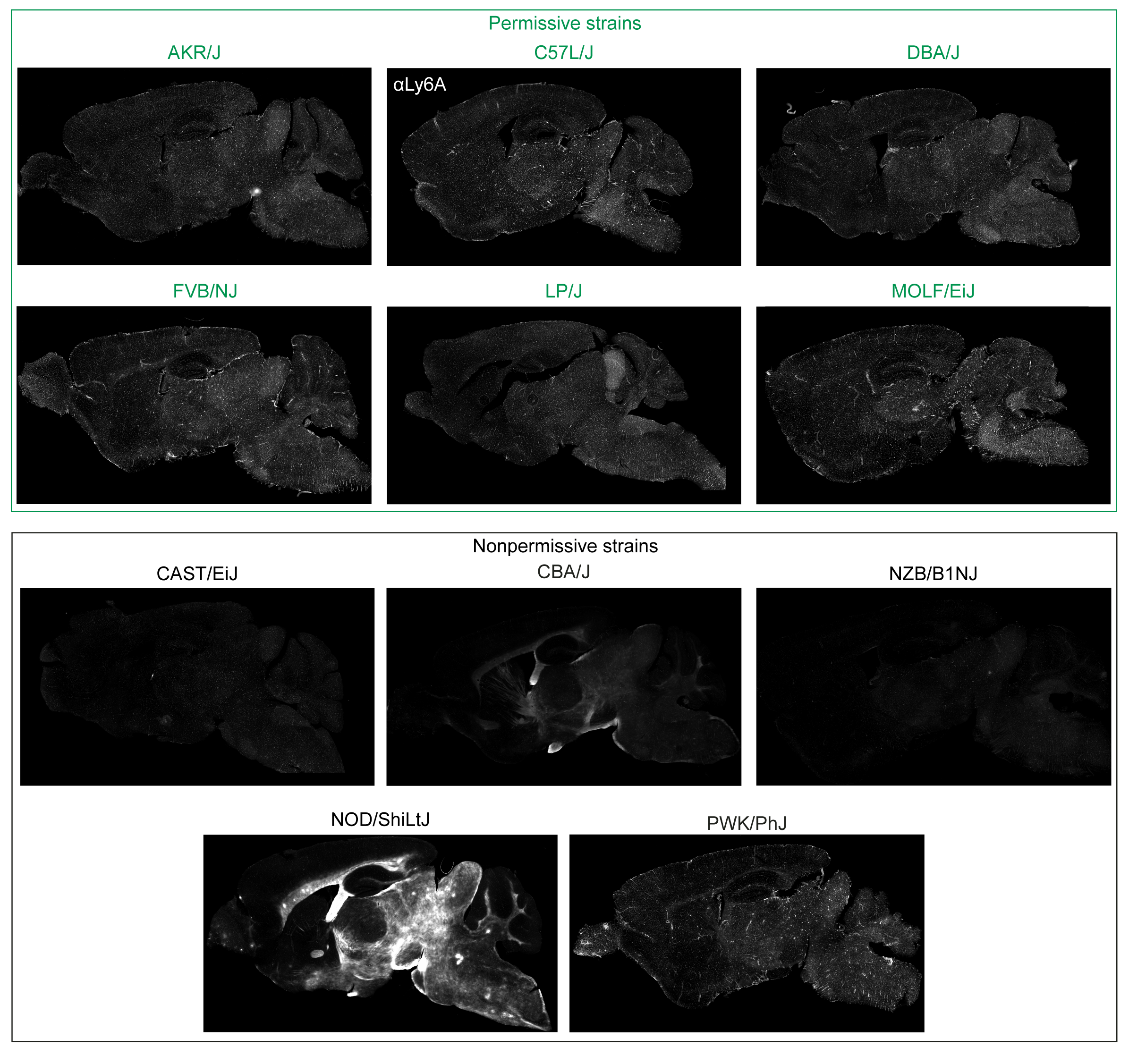

Supplement: S2 Fig — Sagittal whole brain images show LY6A IHC in several representative permissive and nonpermissive mouse lines. (TIF) [file pone.0225206.s005.tif]

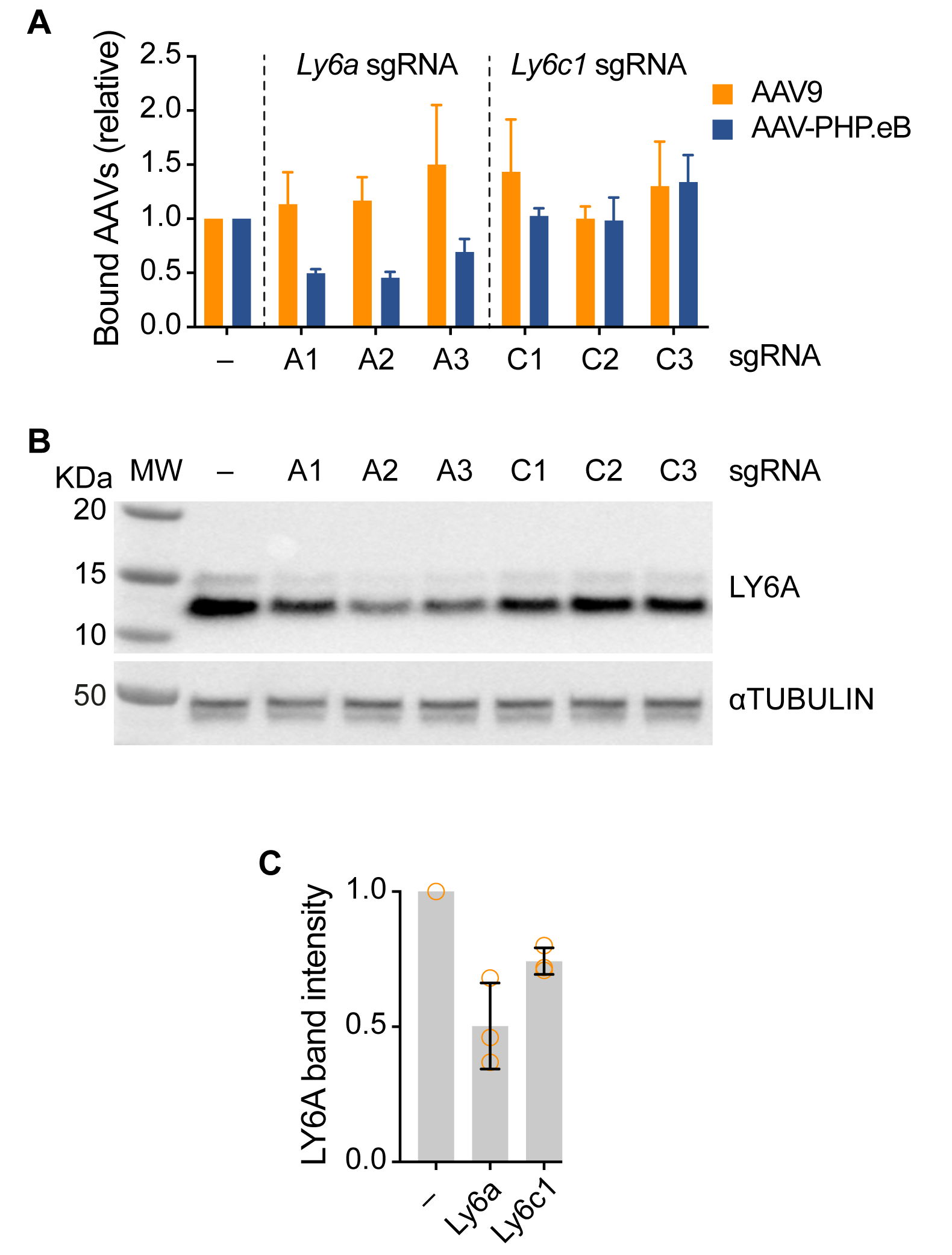

Supplement: S3 Fig — (A) The individual sgRNA data used to generate Fig 3D. (B) Western blots for LY6A (top) or TUBULIN (bottom) in lysates prepared from BMVECs treated with the individual sgRNAs shown in (A). (C) LY6A Western blot band intensity quantification by densitometry. (TIF) [file pone.0225206.s006.tif]

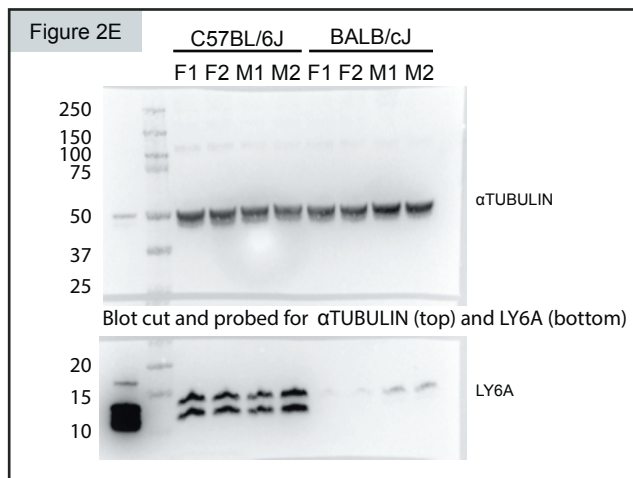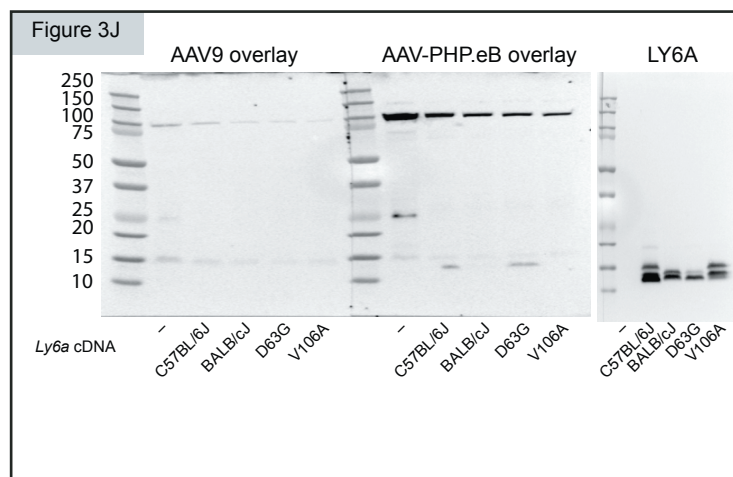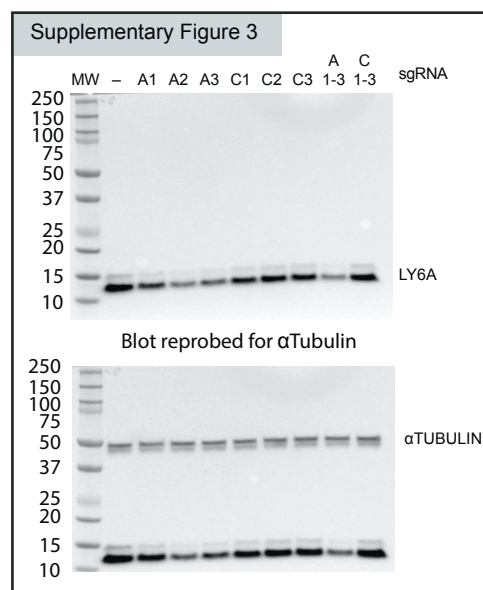

Blots imaged with a BioRad ChemiDoc MP  
BioRad Precision Plus Protein Standard (Dual Color)

Supplement: S3 File — (PDF) [file pone.0225206.s010.pdf]
