# Supplementary material for: Delivering genes across the blood-brain barrier: LY6A, a novel cellular receptor for AAV-PHP.B capsids
Source: PLoS One. 2019 Nov 14;14(11):e0225206. doi: 10.1371/journal.pone.0225206 (PMC6855452; doi:10.1371/journal.pone.0225206)
Supplement: S4 Fig — The schematic on the left shows the structure of the exons and introns of the Ly6a isoform 1. The genetic variants present in at least one of the nonpermissive strains (column headers in blue text) but not the permissive strains (column headers in green) are shown. Variants that segregate between permissive and nonpermissive strains are highlighted in orange text. Variants within exons are highlighted in gray. Variants are shown as homozygous (2), heterozygous (1), absent (0), or as data not available (-). (PDF) [file pone.0225206.s007.pdf]

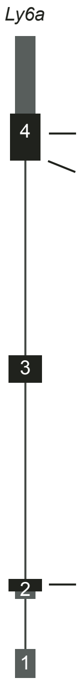

locus	csq terms	alleles	BALB/cJ	CBA/J	NOD/ShiLtJ	NZB/B1NJ	CAST/EiJ	PWK/PhJ	AKR/J	C57L/J	DBA/2J	FVB/NJ	LP/J	MOLF/EiJ	NZW/LacJ
15:74990018	downstream gene variant	["CCT","C"]	-	-	2	2	-	-	-	-	-	-	-	-	-
15:74990020	downstream gene variant	["T","C"]	0	2	0	0	2	0	0	0	0	0	0	0	0
15:74990041	downstream gene variant	["G","GC"]	2	2	2	2	2	2	-	-	-	-	-	-	-
15:74990065	downstream gene variant	["T","TTC"]	-	-	1	1	2	2	-	-	-	-	-	-	-
15:74990099	downstream gene variant	["C","A"]	0	0	0	0	0	2	0	0	0	0	0	0	0
15:74990144	downstream gene variant	["A","G"]	0	0	0	0	1	0	0	0	0	0	0	0	0
15:74990164	downstream gene variant	["A","ATCT"]	-	-	-	1	-	-	-	-	-	-	-	-	-
15:74990173	downstream gene variant	["G","C"]	0	0	1	0	0	0	0	0	0	0	0	0	0
15:74990199	downstream gene variant	["T","A"]	2	2	1	1	0	0	0	0	0	0	0	0	0
15:74990289	downstream gene variant	["A","C"]	0	0	1	0	0	0	0	0	0	0	0	0	0
15:74990290	downstream gene variant	["C","T"]	0	0	1	0	0	0	0	0	0	0	0	0	0
15:74990299	downstream gene variant	["G","A"]	2	2	1	2	0	0	0	0	0	0	0	0	0
15:74990425	downstream gene variant	["C","T"]	2	2	2	2	0	0	0	0	0	0	0	0	0
15:74990438	downstream gene variant	["GC","G"]	2	2	1	1	-	-	-	-	-	-	-	-	-
15:74990469	downstream gene variant	["G","T"]	2	2	2	2	0	0	0	0	0	0	0	0	0
15:74990492	downstream gene variant	["A","G"]	2	2	2	2	0	0	0	0	0	0	0	0	0
15:74990505	downstream gene variant	["C","A"]	2	2	1	1	0	0	0	0	0	0	0	0	0
15:74990642	downstream gene variant	["CT","C"]	-	-	-	-	-	2	-	-	-	-	-	-	-
15:74990643	downstream gene variant	["T","TCC"]	-	-	-	-	2	-	-	-	-	-	-	-	-
15:74990698	downstream gene variant	["A","ATCTCTC"]	0	0	0	-	2	-	0	-	-	-	-	0	-
15:74990698	downstream gene variant	["ATCTCTCTC","A"]	0	0	1	-	0	-	0	-	-	-	-	0	-
15:74990698	downstream gene variant	["ATCTCTCTCTCTCTC","A"]	2	2	0	-	0	-	0	-	-	-	-	0	-
15:74990738	downstream gene variant	["C","T"]	2	2	0	2	0	2	0	0	0	0	0	2	0
15:74990847	downstream gene variant	["TC","T"]	-	-	-	-	2	-	-	-	-	-	-	-	-
15:74990852	downstream gene variant	["T","C"]	0	0	0	0	0	2	0	0	0	0	0	0	0
15:74990859	downstream gene variant	["G","A"]	0	0	0	0	0	2	0	0	0	0	0	0	0
15:74990984	downstream gene variant	["T","C"]	2	2	2	2	2	2	0	0	0	0	0	0	0
15:74991340	downstream gene variant	["A","G"]	0	0	0	0	0	2	0	0	0	0	0	0	0
15:74991607	downstream gene variant	["GGA","G"]	-	0	0	0	2	0	-	-	-	-	-	-	-
15:74991607	downstream gene variant	["G","GGAGAGAGA"]	-	1	0	0	0	0	-	-	-	-	-	-	-
15:74991607	downstream gene variant	["GGAGAGAGAGA","G"]	-	0	0	0	0	2	-	-	-	-	-	-	-
15:74991674	downstream gene variant	["C","G"]	0	0	0	0	2	0	0	0	0	0	0	0	0
15:74991683	downstream gene variant	["A","AAG"]	-	-	-	-	-	2	-	-	-	-	-	-	-
15:74991693	downstream gene variant	["A","G"]	0	0	0	0	0	2	0	0	0	0	0	0	0
15:74991725	downstream gene variant	["AG","A"]	-	-	-	-	-	2	-	-	-	-	-	-	-
15:74991774	downstream gene variant	["ATG","A"]	-	-	-	1	-	-	-	-	-	-	-	-	-
15:74991776	downstream gene variant	["G","A"]	0	-	-	2	-	-	0	0	0	0	0	0	0
15:74991791	downstream gene variant	["C","T"]	-	0	0	1	-	-	0	0	0	0	0	0	0
15:74991807	downstream gene variant	["G","T"]	0	0	0	2	0	2	0	0	0	0	0	0	0
15:74992145	downstream gene variant	["C","T"]	2	1	0	0	0	0	0	0	0	0	0	0	0
15:74992209	downstream gene variant	["G","T"]	0	1	0	0	0	0	0	0	0	0	0	0	0
15:74992261	downstream gene variant	["T","G"]	0	1	0	0	0	0	0	0	0	0	0	0	0
15:74992357	downstream gene variant	["T","G"]	0	0	0	0	1	0	0	0	0	0	0	0	0
15:74992491	downstream gene variant	["C","T"]	0	0	1	1	2	0	0	0	0	0	0	0	0
15:74992498	downstream gene variant	["T","C"]	0	0	1	1	2	0	0	0	0	0	0	0	0
15:74992505	downstream gene variant	["G","C"]	0	0	1	1	2	2	0	0	0	0	0	0	0
15:74992542	downstream gene variant	["C","T"]	0	0	1	1	2	0	0	0	0	0	0	0	0
15:74992569	downstream gene variant	["G","A"]	0	0	1	1	2	0	0	0	0	0	0	0	0
15:74992570	downstream gene variant	["T","A"]	0	0	1	1	2	0	0	0	0	0	0	0	0
15:74992595	downstream gene variant	["G","T"]	0	0	1	1	2	0	0	0	0	0	0	0	0
15:74992602	downstream gene variant	["C","T"]	0	0	1	1	2	2	0	0	0	0	0	0	0
15:74992625	downstream gene variant	["C","G"]	2	2	2	2	2	2	0	0	0	0	0	0	0
15:74992642	downstream gene variant	["C","T"]	0	0	1	1	2	2	0	0	0	0	0	0	0
15:74992675	downstream gene variant	["T","C"]	0	0	1	1	2	2	0	0	0	0	0	0	0
15:74992677	downstream gene variant	["G","T"]	0	0	1	1	2	2	0	0	0	0	0	0	0
15:74992997	downstream gene variant	["G","A"]	2	2	1	1	0	0	0	0	0	0	0	0	0
15:74993197	downstream gene variant	["A","G"]	0	0	0	0	0	2	0	0	0	0	0	0	0
15:74993238	downstream gene variant	["A","G"]	0	0	0	0	0	2	0	0	0	0	0	0	0
15:74993239	downstream gene variant	["G","C"]	0	0	0	0	0	2	0	0	0	0	0	0	0
15:74993298	downstream gene variant	["A","G"]	2	2	2	2	2	2	0	0	0	0	0	2	0
15:74993317	downstream gene variant	["T","G"]	0	0	0	0	0	2	0	0	0	0	0	0	0
15:74993489	downstream gene variant	["G","C"]	0	0	0	0	2	0	0	0	0	0	0	0	0
15:74993509	downstream gene variant	["A","C"]	0	0	0	0	2	0	0	0	0	0	0	0	0
15:74993519	downstream gene variant	["C","A"]	0	0	0	0	0	2	0	0	0	0	0	0	0
15:74993586	downstream gene variant	["A","G"]	2	2	1	1	0	0	0	0	0	0	0	0	0
15:74993792	downstream gene variant	["G","C"]	0	0	0	0	2	0	0	0	0	0	0	0	0
15:74993799	downstream gene variant	["A","C"]	0	0	1	1	2	2	0	0	0	0	0	0	0
15:74993839	downstream gene variant	["G","A"]	0	0	0	0	2	0	0	0	0	0	0	0	0
15:74993851	downstream gene variant	["TC","T"]	-	-	-	-	2	-	-	-	-	-	-	-	-
15:74993855	downstream gene variant	["A","ATC"]	-	-	-	-	2	-	-	-	-	-	-	-	-
15:74993977	downstream gene variant	["C","A"]	2	2	2	2	2	2	0	0	0	0	0	2	0
15:74994138	downstream gene variant	["G","T"]	0	0	0	0	2	0	0	0	0	0	0	0	0
15:74994155	downstream gene variant	["G","A"]	0	0	0	0	2	0	0	0	0	0	0	0	0
15:74994209	downstream gene variant	["G","A"]	0	0	0	0	0	2	0	0	0	0	0	0	0
15:74994289	downstream gene variant	["G","C"]	2	2	2	2	0	0	0	0	0	0	0	0	0
15:74994322	downstream gene variant	["G","A"]	0	0	0	0	2	0	0	0	0	0	0	0	0
15:74994500	downstream gene variant	["AGG","A"]	2	2	2	2	2	2	-	-	-	-	-	2	-
15:74994729	downstream gene variant	["T","G"]	2	2	1	1	0	0	0	0	0	0	0	0	0
15:74994790	downstream gene variant	["A","G"]	0	0	0	0	2	2	0	0	0	0	0	0	0
15:74995070	3' prime UTR variant	["C","A"]	0	0	0	0	0	2	0	0	0	0	0	0	0
15:74995074	3' prime UTR variant	["C","T"]	0	0	0	0	2	0	0	0	0	0	0	0	0
15:74995098	3' prime UTR variant	["A","G"]	0	0	0	0	2	0	0	0	0	0	0	0	0
15:74995271	synonymous variant	["G","A"]	0	0	0	0	0	2	0	0	0	0	0	0	0
15:74995350	**missense variant**	**["A","G"], V106A**	2	2	2	2	2	2	0	0	0	0	0	0	0
15:74995379	synonymous variant	["A","C"]	0	0	0	0	0	2	0	0	0	0	0	0	0
15:74995388	synonymous variant	["G","A"]	2	2	2	2	0	0	0	0	0	0	0	0	0
15:74995479	**missense variant, splice region variant**	**["T","C"], D63G**	2	2	2	2	0	0	0	0	0	0	0	0	0
15:74995537	intron variant	["C","T"]	0	0	0	0	2	0	0	0	0	0	0	0	0
15:74995674	intron variant	["C","CACATCCTGGTTT"]	2	2	-	-	2	2	-	-	-	-	-	2	-
15:74995676	intron variant	["C","CATCCTGGTTTAT"]	-	-	2	2	-	-	-	-	-	-	-	-	-
15:74996019	intron variant	["T","A"]	2	2	2	2	2	2	0	0	0	0	0	0	0
15:74996100	intron variant	["T","TTCCTTCAGCCTAAAC"]	2	2	2	2	2	2	-	-	-	-	-	2	-
15:74996153	intron variant	["A","G"]	2	2	2	2	2	2	0	0	0	0	0	2	0
15:74996362	intron variant	["G","T"]	0	0	0	0	2	0	0	0	0	0	0	0	0
15:74996365	intron variant	["G","T"]	0	0	0	0	0	2	0	0	0	0	0	0	0
15:74996688	intron variant	["G","C"]	0	0	0	0	2	0	0	0	0	0	0	0	0
15:74996783	intron variant	["A","G"]	2	2	2	2	0	0	0	0	0	0	0	0	0
15:74996807	intron variant	["A","G"]	0	0	0	0	2	0	0	0	0	0	0	0	0
15:74996836	intron variant	["C","G"]	0	0	0	0	2	2	0	0	0	0	0	0	0
15:74996872	intron variant	["G","T"]	0	0	0	0	2	0	0	0	0	0	0	0	0
15:74996926	intron variant	["C","T"]	0	0	0	0	1	0	0	0	0	0	0	0	0
15:74996931	intron variant	["C","T"]	0	0	0	0	1	0	0	0	0	0	0	0	0
15:74996936	intron variant	["G","T"]	0	0	0	0	1	0	0	0	0	0	0	0	0
15:74996944	intron variant	["A","T"]	0	0	0	0	1	0	0	0	0	0	0	0	0
15:74996953	intron variant	["A","G"]	0	0	0	0	1	0	0	0	0	0	0	0	0
15:74996959	intron variant	["A","T"]	0	0	0	0	1	0	0	0	0	0	0	0	0
15:74997126	intron variant	["G","A"]	0	0	0	0	1	0	0	0	0	0	0	0	0
15:74997169	intron variant	["G","A"]	0	0	0	0	1	0	0	0	0	0	0	0	0
15:74997170	intron variant	["G","A"]	0	0	0	0	1	0	0	0	0	0	0	0	0
15:74997220	intron variant	["T","C"]	2	2	2	2	0	2	0	0	0	0	0	0	0
15:74997267	intron variant	["G","A"]	0	0	0	0	2	2	0	0	0	0	0	0	0
15:74997302	intron variant	["C","A"]	2	2	2	2	2	2	0	0	0	0	0	0	0
15:74997369	intron variant	["A","T"]	2	2	2	2	2	2	0	0	0	0	0	0	0
15:74997400	intron variant	["G","A"]	2	2	2	2	1	2	0	0	0	0	0	0	0
15:74997403	intron variant	["C","A"]	2	2	2	2	1	2	0	0	0	0	0	0	0
15:74997412	intron variant	["T","C"]	0	1	0	0	0	0	0	0	0	0	0	0	0
15:74997574	**missense variant**	**["A","C"], L11V**	0	1	0	0	2	2	0	0	0	0	0	0	0
15:74997610	5' UTR variant	["G","C"]	0	0	0	0	1	0	0	0	0	0	0	0	0
15:74997632	intron variant	["C","T"]	0	0	0	0	1	0	0	0	0	0	0	0	0
15:74997650	intron variant	["A","G"]	2	2	2	2	2	2	0	0	0	0	0	0	0
15:74997687	intron variant	["G","C"]	2	2	1	2	1	2	0	0	0	0	0	0	0
15:74997688	intron variant	["A","G"]	2	1	2	2	1	2	0	0	0	0	0	2	0
15:74997694	intron variant	["G","A"]	0	1	0	0	0	0	0	0	0	0	0	0	0
15:74997695	intron variant	["G","C"]	2	1	1	2	2	2	0	0	0	0	0	1	0
15:74997700	intron variant	["C","A"]	0	0	0	0	0	1	0	0	0	0	0	0	0
15:74997719	intron variant	["C","G"]	0	0	0	0	1	0	0	0	0	0	0	0	0
15:74997721	intron variant	["CT","C"]	-	-	-	-	2	2	-	-	-	-	-	-	-
15:74997724	intron variant	["C","T"]	2	2	2	2	0	1	0	0	0	0	0	0	0
15:74997738	intron variant	["T","C"]	0	0	0	0	1	0	0	0	0	0	0	0	0
15:74997739	intron variant	["G","A"]	0	0	0	0	1	0	0	0	0	0	0	0	0
15:74997780	intron variant	["C","A"]	1	0	0	0	0	0	0	0	0	0	0	0	0
15:74997786	intron variant	["C","T"]	0	0	0	0	1	0	0	0	0	0	0	0	0
15:74997812	intron variant	["C","A"]	0	0	0	0	0	2	0	0	0	0	0	0	0
15:74997831	intron variant	["G","A"]	0	0	0	0	1	0	0	0	0	0	0	0	0
15:74997850	intron variant	["A","C"]	1	1	2	1	0	0	0	0	0	0	0	0	0

locus	csq terms	alleles	BALB/cJ	CBA/J	NOD/ShiLtJ	NZB/B1NJ	CAST/EiJ	PWK/PhJ	AKR/J	C57L/J	DBA/2J	FVB/NJ	LP/J	MOLF/EiJ	NZW/LacJ
15:74997851	intron variant	["G","A"]	1	1	2	2	0	0	0	0	0	0	0	0	0
15:74997864	intron variant	["C","G"]	0	1	0	0	0	2	0	0	0	0	0	0	0
15:74997894	5' UTR variant	["G","A"]	0	0	1	0	0	0	0	0	0	0	0	0	0
15:74998047	upstream gene variant	["T","C"]	0	0	1	1	0	2	0	0	0	0	0	0	0
15:74998055	upstream gene variant	["C","A"]	0	0	0	0	0	2	0	0	0	0	0	0	0
15:74998095	upstream gene variant	["G","A"]	1	1	1	1	2	2	0	0	0	0	0	0	0
15:74998096	upstream gene variant	["T","G"]	1	1	1	1	2	2	0	0	0	0	0	0	0
15:74998117	upstream gene variant	["G","A"]	0	0	0	0	0	2	0	0	0	0	0	0	0
15:74998122	upstream gene variant	["C","G"]	1	1	2	1	2	2	0	0	0	0	0	0	0
15:74998192	upstream gene variant	["A","C"]	2	2	2	2	2	2	0	0	0	0	0	0	0
15:74998217	upstream gene variant	["G","A"]	0	0	0	0	0	2	0	0	0	0	0	0	0
15:74998228	upstream gene variant	["T","C"]	2	2	2	2	1	0	0	0	0	0	0	0	0
15:74998228	upstream gene variant	["T","A"]	0	0	0	0	0	2	0	0	0	0	0	0	0
15:74998240	upstream gene variant	["G","GTTTTTAT"]	-	-	-	-	2	-	-	-	-	-	-	-	-
15:74998244	upstream gene variant	["T","TTATTTTTA"]	2	2	2	2	-	-	-	-	-	-	-	-	-
15:74998245	upstream gene variant	["T","TA"]	-	-	-	-	-	2	-	-	-	-	-	-	-
15:74998251	upstream gene variant	["G","T"]	0	0	0	0	0	2	0	0	0	0	0	0	0
15:74998252	upstream gene variant	["A","C"]	0	2	2	2	2	0	0	0	0	0	0	0	0
15:74998253	upstream gene variant	["C","T"]	0	0	0	0	0	2	0	0	0	0	0	0	0
15:74998258	upstream gene variant	["T","TTGCC"]	-	-	-	-	-	2	-	-	-	-	-	-	-
15:74998260	upstream gene variant	["T","C"]	0	0	0	0	1	0	0	0	0	0	0	0	0
15:74998262	upstream gene variant	["T","TGCC"]	-	-	-	-	-	2	-	-	-	-	-	-	-
15:74998272	upstream gene variant	["G","T"]	0	0	0	1	0	0	0	0	0	0	0	0	0
15:74998298	upstream gene variant	["C","T"]	2	2	2	1	2	2	0	0	0	0	0	0	0
15:74998313	upstream gene variant	["C","A"]	2	2	2	2	2	2	0	0	0	0	0	0	0
15:74998322	upstream gene variant	["A","C"]	2	2	2	2	2	2	0	0	0	0	0	2	0
15:74998395	upstream gene variant	["C","G"]	0	0	0	0	1	0	0	0	0	0	0	0	0
15:74998406	upstream gene variant	["G","T"]	2	2	2	2	1	2	0	0	0	0	0	0	0
15:74998469	upstream gene variant	["A","C"]	2	2	2	2	2	0	0	0	0	0	0	2	0
15:74998485	upstream gene variant	["A","C"]	2	2	2	1	1	0	0	0	0	0	0	0	0
15:74998487	upstream gene variant	["T","C"]	2	2	2	2	2	2	0	0	0	0	0	0	0
15:74998627	upstream gene variant	["T","G"]	2	2	2	2	2	2	0	0	0	0	0	0	0
15:74998720	upstream gene variant	["A","C"]	2	2	2	2	0	1	0	0	0	0	0	0	0
15:74998750	upstream gene variant	["C","T"]	0	0	0	0	1	0	0	0	0	0	0	0	0
15:74998809	upstream gene variant	["T","C"]	2	2	2	2	0	0	0	0	0	0	0	0	0
15:74998866	upstream gene variant	["A","T"]	0	0	0	0	1	0	0	0	0	0	0	0	0
15:74998893	upstream gene variant	["A","G"]	2	2	2	2	2	2	0	0	0	0	0	0	0
15:74998949	upstream gene variant	["C","A"]	2	2	2	2	0	1	0	0	0	0	0	0	0
15:74998952	upstream gene variant	["T","C"]	2	2	2	2	0	1	0	0	0	0	0	0	0
15:74999052	upstream gene variant	["A","T"]	2	0	0	0	0	-	0	0	0	0	0	0	0
15:74999173	upstream gene variant	["C","A"]	0	0	0	0	2	0	0	0	0	0	0	0	0
15:74999213	upstream gene variant	["C","A"]	0	0	0	0	2	0	0	0	0	0	0	0	0
15:74999262	upstream gene variant	["C","A"]	2	2	2	2	-	2	0	0	0	0	0	0	0
15:74999426	upstream gene variant	["G","A"]	2	2	2	2	0	0	0	0	0	0	0	0	0
15:74999451	upstream gene variant	["G","C"]	2	0	0	2	0	2	0	0	0	0	0	0	0
15:74999507	upstream gene variant	["A","G"]	2	2	2	2	0	2	0	0	0	0	0	2	0
15:74999531	upstream gene variant	["C","A"]	0	0	0	0	0	2	0	0	0	0	0	0	0
15:74999541	upstream gene variant	["C","A"]	0	0	0	0	2	0	0	0	0	0	0	0	0
15:74999553	upstream gene variant	["G","A"]	2	2	2	2	0	0	0	0	0	0	0	0	0
15:74999577	upstream gene variant	["C","A"]	0	0	0	0	2	0	0	0	0	0	0	0	0
15:74999642	upstream gene variant	["G","A"]	0	0	0	0	2	0	0	0	0	0	0	0	0
15:74999809	upstream gene variant	["C","T"]	1	1	2	2	0	0	0	0	0	0	0	0	0
15:74999818	upstream gene variant	["T","G"]	0	0	0	0	0	2	0	0	0	0	0	0	0
15:74999900	upstream gene variant	["C","A"]	2	2	2	2	2	0	0	0	0	0	0	0	0
15:74999931	upstream gene variant	["C","G"]	2	2	2	2	0	0	0	0	0	0	0	0	0
15:74999957	upstream gene variant	["C","T"]	1	1	2	2	0	0	0	0	0	0	0	0	0
15:74999970	upstream gene variant	["GGATGCA","G"]	-	-	-	-	1	-	-	-	-	-	-	-	-
15:75000010	upstream gene variant	["A","G"]	2	2	2	2	2	2	0	0	0	0	0	0	0
15:75000013	upstream gene variant	["C","T"]	1	0	0	0	0	0	0	0	0	0	0	0	0
15:75000044	upstream gene variant	["T","C"]	2	2	2	2	2	2	0	0	0	0	0	1	0
15:75000064	upstream gene variant	["A","C"]	0	0	0	0	0	1	0	0	0	0	0	0	0
15:75000068	upstream gene variant	["GC","G"]	0	1	-	1	2	2	-	-	-	-	-	1	-
15:75000126	upstream gene variant	["A","G"]	0	0	0	0	1	0	0	0	0	0	0	0	0
15:75000194	upstream gene variant	["C","A"]	1	1	0	1	2	2	0	0	0	0	0	0	0
15:75000198	upstream gene variant	["G","C"]	1	1	0	1	2	2	0	0	0	0	0	0	0
15:75000236	upstream gene variant	["A","G"]	0	0	0	0	1	0	0	0	0	0	0	0	0
15:75000268	upstream gene variant	["A","T"]	0	0	0	0	1	0	0	0	0	0	0	1	0
15:75000325	upstream gene variant	["G","C"]	0	0	0	0	0	1	0	0	0	0	0	0	0
15:75000337	upstream gene variant	["G","A"]	1	1	2	1	0	0	0	0	0	0	0	0	0
15:75000342	upstream gene variant	["A","G"]	1	1	2	2	0	1	0	0	0	0	0	0	0
15:75000383	upstream gene variant	["T","C"]	2	2	2	2	0	2	0	0	0	0	0	2	0
15:75000396	upstream gene variant	["C","T"]	2	2	2	2	0	0	0	0	0	0	0	0	0
15:75000405	upstream gene variant	["T","C"]	2	2	2	2	0	0	0	0	0	0	0	0	0
15:75000532	upstream gene variant	["C","G"]	2	2	2	2	0	0	0	0	0	0	0	0	0
15:75000538	upstream gene variant	["C","A"]	2	2	2	2	0	0	0	0	0	0	0	0	0
15:75000539	upstream gene variant	["A","C"]	2	2	2	2	0	0	0	0	0	0	0	0	0
15:75000563	upstream gene variant	["A","C"]	0	0	0	0	0	2	0	0	0	0	0	0	0
15:75000593	upstream gene variant	["A","C"]	0	0	0	0	-	2	0	0	0	0	0	0	0
15:75000594	upstream gene variant	["G","A"]	0	0	0	0	-	2	0	0	0	0	0	0	0
15:75000632	upstream gene variant	["A","T"]	0	0	0	0	0	2	0	0	0	0	0	0	0
15:75000707	upstream gene variant	["A","G"]	0	0	0	0	0	2	0	0	0	0	0	0	0
15:75000718	upstream gene variant	["C","T"]	2	2	2	2	0	0	0	0	0	0	0	0	0
15:75000723	upstream gene variant	["C","T"]	2	2	2	2	0	0	0	0	0	0	0	0	0
15:75000801	upstream gene variant	["C","A"]	2	2	2	2	0	2	0	0	0	0	0	0	0
15:75000809	upstream gene variant	["C","A"]	2	2	2	2	-	0	0	0	0	0	0	0	0
15:75000843	upstream gene variant	["G","T"]	0	2	0	2	-	0	0	0	0	0	0	0	0
15:75000860	upstream gene variant	["G","T"]	2	2	2	2	-	0	0	0	0	0	0	0	0
15:75000865	upstream gene variant	["GT","G"]	2	2	2	2	-	-	-	-	-	-	-	-	-
15:75000890	upstream gene variant	["T","C"]	0	0	0	0	-	2	0	0	0	0	0	0	0
15:75000909	upstream gene variant	["G","C"]	0	0	0	0	0	2	0	0	0	0	0	0	0
15:75000951	upstream gene variant	["T","C"]	2	2	2	2	0	2	0	0	0	0	0	2	0
15:75001009	upstream gene variant	["C","T"]	2	2	2	2	0	0	0	0	0	0	0	0	0
15:75001037	upstream gene variant	["A","G"]	1	2	2	2	0	0	0	0	0	0	0	0	0
15:75001076	upstream gene variant	["C","T"]	1	0	0	0	2	0	0	0	0	0	0	0	0
15:75001077	upstream gene variant	["G","A"]	1	2	2	2	0	0	0	0	0	0	0	0	0
15:75001080	upstream gene variant	["C","A"]	1	2	2	2	0	0	0	0	0	0	0	0	0
15:75001081	upstream gene variant	["C","T"]	1	2	2	2	0	0	0	0	0	0	0	0	0
15:75001117	upstream gene variant	["G","A"]	2	2	2	2	0	0	0	0	0	0	0	0	0
15:75001153	upstream gene variant	["T","C"]	0	0	0	0	2	0	0	0	0	0	0	0	0
15:75001281	upstream gene variant	["T","C"]	2	2	2	2	0	0	0	0	0	0	0	0	0
15:75001330	upstream gene variant	["A","G"]	2	2	2	2	2	0	0	0	0	0	0	0	0
15:75001458	upstream gene variant	["A","C"]	2	2	1	0	0	1	0	0	0	0	0	1	0
15:75001473	upstream gene variant	["G","A"]	0	0	0	0	0	1	0	0	0	0	0	1	0
15:75001591	upstream gene variant	["C","T"]	2	2	2	2	2	2	0	0	0	0	0	0	0
15:75001601	upstream gene variant	["A","G"]	2	2	2	2	2	0	0	0	0	0	0	0	0
15:75001659	upstream gene variant	["A","AC"]	-	-	-	-	2	-	-	-	-	-	-	-	-
15:75001674	upstream gene variant	["G","A"]	2	2	2	2	0	2	0	0	0	0	0	0	0
15:75001692	upstream gene variant	["A","G"]	0	0	0	0	0	2	0	0	0	0	0	0	0
15:75001708	upstream gene variant	["T","C"]	2	2	2	2	0	2	0	0	0	0	0	0	0
15:75001753	upstream gene variant	["A","T"]	1	1	2	2	0	0	0	0	0	0	0	0	0
15:75001761	upstream gene variant	["G","A"]	0	0	0	0	1	0	0	0	0	0	0	0	0
15:75001773	upstream gene variant	["G","A"]	0	0	0	0	2	2	0	0	0	0	0	0	0
15:75001798	upstream gene variant	["A","G"]	2	2	2	2	0	2	0	0	0	0	0	0	0
15:75001816	upstream gene variant	["T","G"]	2	2	2	2	0	0	0	0	0	0	0	0	0
15:75001835	upstream gene variant	["G","A"]	0	0	0	0	1	0	0	0	0	0	0	0	0
15:75001846	upstream gene variant	["GC","G"]	-	-	-	-	1	-	-	-	-	-	-	-	-
15:75002008	upstream gene variant	["T","G"]	1	2	2	2	-	2	0	0	0	0	0	0	0
15:75002061	upstream gene variant	["C","T"]	1	0	0	0	-	0	0	0	0	0	0	0	0
15:75002068	upstream gene variant	["G","T"]	1	0	0	0	-	0	0	0	0	0	0	0	0
15:75002190	upstream gene variant	["C","G"]	2	2	2	2	-	0	0	0	0	0	0	0	0
15:75002191	upstream gene variant	["CCAAAACAAAACAAAACAAAA","C"]	0	-	0	0	-	2	-	-	-	-	-	0	-
15:75002191	upstream gene variant	["CCAAAACAAAACAAAACAAAACAAAA","C"]	2	-	2	2	-	0	-	-	-	-	-	0	-
15:75002460	upstream gene variant	["G","T"]	2	2	2	2	-	0	0	0	0	0	0	0	0
15:75002504	upstream gene variant	["C","T"]	2	2	2	2	-	0	0	0	0	0	0	0	0
15:75002593	upstream gene variant	["A","G"]	2	2	2	2	-	0	0	0	0	0	0	0	0
15:75002648	upstream gene variant	["T","A"]	2	2	2	2	-	0	0	0	0	0	0	0	0
15:75002674	upstream gene variant	["G","C"]	2	2	2	2	-	0	0	0	0	0	0	0	0
15:75002783	upstream gene variant	["A","C"]	2	2	2	2	-	0	0	0	0	0	0	0	0
15:75002795	upstream gene variant	["A","G"]	2	2	2	2	-	0	0	0	0	0	0	0	0
15:75002853	upstream gene variant	["T","A"]	2	2	2	2	-	0	0	0	0	0	0	0	0
15:75002868	upstream gene variant	["C","T"]	0	0	0	0	0	2	0	0	0	0	0	0	0
15:75002870	upstream gene variant	["A","C"]	2	2	2	2	0	0	0	0	0	0	0	0	0
15:75002916	upstream gene variant	["G","A"]	2	2	2	2	0	0	0	0	0	0	0	0	0
15:75002940	upstream gene variant	["A","T"]	2	2	2	2	-	0	0	0	0	0	0	0	0
