# Supplementary material for: Delivering genes across the blood-brain barrier: LY6A, a novel cellular receptor for AAV-PHP.B capsids
Source: PLoS One. 2019 Nov 14;14(11):e0225206. doi: 10.1371/journal.pone.0225206 (PMC6855452; doi:10.1371/journal.pone.0225206)
Supplement: S2 File — (PDF) [file pone.0225206.s009.pdf]

Supplementary References:

1. Keane TM, Goodstadt L, Danecek P, White MA, Wong K, Yalcin B, et al. Mouse genomic variation and its effect on phenotypes and gene regulation. Nature. 2011;477: 289–294.

2. Yalcin B, Wong K, Agam A, Goodson M, Keane TM, Gan X, et al. Sequence-based characterization of structural variation in the mouse genome. Nature. 2011;477: 326–329.

3. Pierleoni A, Martelli PL, Casadio R. PredGPI: a GPI-anchor predictor. BMC Bioinformatics. 2008;9: 392.

4. Deverman BE, Pravdo PL, Simpson BP, Kumar SR, Chan KY, Banerjee A, et al. Cre-dependent selection yields AAV variants for widespread gene transfer to the adult brain. Nat Biotechnol. 2016;34: 204–209.

5. Chan KY, Jang MJ, Yoo BB, Greenbaum A, Ravi N, Wu W-L, et al. Engineered AAVs for efficient noninvasive gene delivery to the central and peripheral nervous systems. Nat Neurosci. 2017;20: 1172–1179.

6. Zelikowsky M, Hui M, Karigo T, Choe A, Yang B, Blanco MR, et al. The Neuropeptide Tac2 Controls a Distributed Brain State Induced by Chronic Social Isolation Stress. Cell. 2018;173: 1265–1279.e19.

7. Allen WE, Kauvar IV, Chen MZ, Richman EB, Yang SJ, Chan K, et al. Global Representations of Goal-Directed Behavior in Distinct Cell Types of Mouse Neocortex. Neuron. 2017;94: 891–907.e6.

8. Hillier D, Fiscella M, Drinnenberg A, Trenholm S, Rompani SB, Raics Z, et al. Causal evidence for retina-dependent and -independent visual motion computations in mouse cortex. Nat Neurosci. 2017;20: 960–968.

9. Hordeaux J, Wang Q, Katz N, Buza EL, Bell P, Wilson JM. The Neurotropic Properties of AAV-PHP.B Are Limited to C57BL/6J Mice. Mol Ther. 2018;26: 664–668.
